# Acupuncture-Related Therapies for Parkinson's Disease: A Meta-Analysis and Qualitative Review

**DOI:** 10.3389/fnagi.2021.676827

**Published:** 2021-07-01

**Authors:** Xiaopeng Wen, Kunbin Li, Hao Wen, Qian Wang, Zhiyuan Wu, Xianli Yao, Bing Jiao, Pingge Sun, Shuqi Ge, Chenyang Wen, Liming Lu

**Affiliations:** ^1^Department of Neurological Rehabilitation, Zhengzhou Central Hospital Affiliated to Zhengzhou University, Zhengzhou, China; ^2^Department of Neurology, Sun Yat-sen Memorial Hospital, Sun Yat-sen University, Guangzhou, China; ^3^Department of Medical Imaging, Zhengzhou Central Hospital Affiliated to Zhengzhou University, Zhengzhou, China; ^4^South China Research Center for Acupuncture and Moxibustion, Medical College of Acu-Moxi and Rehabilitation, Guangzhou University of Chinese Medicine, Guangzhou, China; ^5^Department of Rehabilitation Medicine, Dengfeng City Second People' s Hospital, Zhengzhou, China; ^6^Evidence-Based Medicine and Data Science Centre, Guangzhou University of Chinese Medicine, Guangzhou, China

**Keywords:** qualitative review, meta-analysis, Parkinson's disease, conventional medication, acupuncture-related therapies

## Abstract

**Objective:** This systematic review and meta-analysis aimed to assess the effects of the combination of acupuncture-related therapies with conventional medication compared with conventional medication in patients with Parkinson's disease (PD).

**Methods:** A literature search within eight databases [including Medline, Embase, the Cochrane Library, PubMed, China National Knowledge Infrastructure (CNKI), China Biology Medicine (CBM), VIP, and Wanfang Database] was performed covering a time frame from their inception to August 2020. Randomized controlled trials (RCTs) comparing acupuncture-related therapies combined with conventional medication vs. conventional medication in patients with PD were eligible. Two authors independently assessed the risk of bias. Assessments were performed with the total and subscales scores of the Unified Parkinson's Disease Rating Scale (UPDRS), 39-item Parkinson's Disease Questionnaire (PDQ-39), the dosage of Madopar, Mini-Mental State Examination (MMSE), and 17-item Hamilton Depression Scale (HAMD). Data were analyzed by adopting the Cochrane Collaboration's RevMan 5.4 (Review Man, Copenhagen, Denmark); and mean effect sizes and 95% confidence intervals were estimated. Tests for heterogeneity were used to assess differences in treatment effects across different types of acupuncture used.

**Results:** Sixty-six trials met the inclusion criteria, of which 61 trials provided data for the meta-analysis. We defined high-quality articles as those with a low risk of bias in four or more domains; and only 10 (15.15%) articles were of high quality. Compared with the controls, acupuncture-related therapies with conventional medication achieved a benefit in the primary outcomes of UPDRS (motor subscore: −3.90, −4.33 to −3.49, *P* < 0.01; total score: −7.37 points, −8.91 to −5.82, *P* < 0.001; activities of daily living subscore: −3.96, −4.96 to −2.95, *P* < 0.01). For the subgroup difference test among the effects of different acupuncture methods, significant differences existed in outcomes with the UPDRS-III, UPDRS-I, UPDRS-IV, and PDQ-39 scores and Madopar dosage, while non-significant differences existed with the UPDRS-total, UPDRS-II, HAMD, and MMSE scores.

**Conclusions:** Acupuncture-related therapies combined with conventional medication may benefit individuals with PD. Our review findings should be considered with caution because of the methodological weaknesses in the included trials. Future, large randomized trials of acupuncture-related therapies for PD with high methodological quality are warranted.

**Systematic Review Registration:** Identifier CRD42021228110.

## Introduction

Parkinson's disease (PD) is a complex neurodegenerative disorder with wide-reaching complications for patients and their families. Traditionally, the management of PD has focused on drug treatment and deep brain stimulations, but neither has been shown to definitively delay the progression of the disorder nor to treat non-motor symptoms (Ghaffari and Kluger, 2014). Questions have been raised about the long-time use of anti-Parkinson drugs since they are considered to increase the potential for complications (Lee and Lim, 2017), such as sleeplessness, depression or anxiety, constipation, and cognitive decline, which can lead to a decline in quality of life (QOL).

Owing to the limitations of conventional treatments, at least 40% of PD patients use one or more forms of alternative treatment in addition to traditional treatments to improve symptoms (Ghaffari and Kluger, 2014). In recent years, many researchers have paid attention to alternatives as adjuvant therapies to improve outcomes for patients (Jiang et al., 2018). Based on a systematic review performed by Kluger (Ghaffari and Kluger, 2014), alternative treatments in PD, such as acupuncture, tai chi, qigong, yoga, music therapy, and vibration therapy, have potential clinical or theoretical benefits for patients. Tomlinson recommended that physiotherapy has short-term benefits in ameliorating motor function and improving the QOL of patients (Tomlinson et al., 2012). Among these adjuvant therapies, acupuncture is the most commonly used in patients with PD (Lee and Lim, 2017; Noh et al., 2017; Jiang et al., 2018).

Although several systematic reviews have investigated the efficacy of acupuncture, previous studies have mostly focused on classical and electroacupuncture, especially electroacupuncture (Jiang et al., 2018). There are few articles discussing the effects of different types of acupuncture in patients with PD. While a variety of definitions of the term acupuncture have been suggested, this paper will adopt the definition suggested by the World Health Organization: acupuncture literally means puncturing with a needle. However, it may also involve the application of other kinds of stimulation to certain points. In this paper, the term acupuncture will be used in its broadest sense to refer to any type of commonly used acupunctures that simulate certain points with needles, lasers, electricity, or pressure. Therefore, the specific types of acupuncture therapies included in this manuscript are traditional body needling, manual acupuncture, electroacupuncture, ear (auricular) acupuncture, auricular pressure, scalp acupuncture, bee venom acupuncture (BVA), abdominal acupuncture, acupoint catgut embedding, and acupressure. Acupuncture-related therapies include forms combined with moxibustion or medication, such as warm needling, acupoint injection, hydroacupuncture, or herbal decoction.

This review includes trials assessing a variety of different types of acupuncture methods to provide an overall assessment of the use of different types of acupuncture in PD patients.

## Methods

### Data Sources and Search Strategy

For English publications, data were gathered from four separate electronic databases, including Medline (Ovid), Embase (Ovid), the Cochrane Library (Ovid), and PubMed. For Chinese articles, online databases such as the China National Knowledge Infrastructure (CNKI), China Biology Medicine (CBM) disc, Chinese Scientific Journals Full-Text Database (VIP), and Wanfang Database were searched. Searches were confined to publications in the English and Chinese languages. No restrictions were imposed on publication years. The reference lists of the included studies were also manually searched to identify relevant articles. The obtained studies were further evaluated by review of titles and abstracts to exclude those not meeting the criteria, and the full text was then read to determine eligibility. Detailed search strategies are described in **Appendix 1**.

### Study Selection

#### Types of Studies

Randomized controlled trials (RCTs) evaluating acupuncture-related therapies in the treatment of PD were considered, regardless of blinding, publication status, and length of trial. Non-randomized uncontrolled trials were excluded. Additionally, dissertations, cell culture or animal experiments, case reports, case series, conference papers, and editorials were excluded, as were articles published as abstracts only and some types of crossover study designs.

#### Types of Participants

Study participants were patients who received a diagnosis of any type of PD using standard diagnostic criteria, such as the UK Parkinson's Disease Society Brain Bank criteria (Rajput, 1993), the criteria for the Diagnosis and Differential Diagnosis for Parkinson's Disease of the 1984 Chinese National Conference for Extrapyramidal Disease (Wang, 1985), different versions of the Diagnostic Criteria of Parkinson's disease in China (Liu, 2002; Liu J., 2016), and diagnostic criteria for idiopathic PD (Jiang Y. P. et al., 2006). Age, sex, ethnicity, or disease duration restrictions were not applied.

#### Types of Interventions

Studies that involved the use of a combination of acupuncture-related therapies with Chinese medicine (CM) were included. These techniques encompass a wide range, as mentioned above with regard to the definition of acupuncture; the definition of acupuncture-related therapies included classical acupuncture (with or without electrical stimulation, and manual or scalp acupuncture), ear (auricular) acupuncture, auricular pressure, BVA, abdominal acupuncture, acupoint catgut embedding and acupressure, moxibustion (including direct and indirect moxibustion and warm needling), or acupoint injection or herbal decoction.

All the control groups received CM. For this paper, CM was defined as an anti-Parkinson drug, including Madopar, levodopa, carbidopa, and pramipexole, used alone or in combination. If the control group received CM with other medicines or treatments, acupuncture-related therapies would be the only intervention that differed between the experimental and control groups.

The exclusion criteria included the following: (1) the study design did not evaluate the effects of acupuncture-related therapies on PD symptoms; (2) the experimental group consisted of more than three interventions; (3) the study compared different types of acupuncture or interventions; and (4) the study reported insufficient information or incorrect data, or it was a republished article.

#### Types of Outcome Measures

Various outcome measures were used in the selected studies. The Unified Parkinson's Disease Rating Scale (UPDRS) is the most commonly used rating scale and consists of four segments: UPDRS-I (mentation, behavior, and mood); UPDRS-II (activities of daily living); UPDRS-III (motor); and UPDRS-IV (complications) (Asakawa et al., 2016). Scores range from 0 to 199, with higher scores indicating greater disability. The primary outcome indicators included UPDRS-total, UPDRS-II, and UPDRS-III scores.

The secondary outcomes included UPDRS-I, UPDRS-IV, Hamilton Depression Scale (HAMD), and PDQ-39 scores, Madopar dosages, and HAMD and Mini-Mental State Examination (MMSE) scores. We used the 17-item HAMD to measure the severity of depressive symptoms in patients with PD. The total HAMD score can range from 0 to a maximum of 54 points, with higher scores indicating more severe depression. QOL was assessed by the Parkinson's Disease Questionnaire (PDQ-39), which consists of 39 questions distributed across eight domains (including mobility, activities of daily living, emotional well-being, stigma, social support, cognitions, communication, and bodily discomfort). Each item is rated on a 5-point scale (0–4) (Hagell and Nygren, 2007). Lower scores reflect better QOL. The dosages of Madopar were calculated in milligrams; and the lower the dosage, the lower the dependence on the drug. PD-related cognitive impairment was measured by the MMSE, which includes 11 questions (Folstein et al., 1975). Higher scores reflect better cognitive function.

### Data Extraction

First, one reviewer (WXP) identified duplicate literature using Note-express 3.2.0, scanned the titles and abstracts of the literature, and classified the papers into different categories according to inclusion and exclusion criteria (first scan). Then, according to the predefined criteria, two independent reviewers (WXP and WH) read the full texts of all potentially eligible studies. The characteristics of each study (author, publication year, and originating country), patient information (age, sex, number, and disease duration), intervention and control groups (frequency and types of therapy), outcome measures, the results, and adverse events were extracted. The means and standard deviations (mean ± SD) of the primary and secondary outcomes were retrieved from the articles. If data were reported at different time points, those from immediately after the intervention were used. If studies presented insufficient data or uncertain information, we contacted the corresponding authors. Any discrepancies about study inclusion and data extraction were resolved by discussion.

### Quality Assessment

The methodological quality of the studies was assessed by the Cochrane risk of bias assessment (version 5.1.0) (Higgins and Green, 2011). This tool covers seven domains: random sequence generation; allocation concealment; blinding of patients, personnel, and outcome assessors; incomplete outcome data; selective outcome reporting; and other sources of bias. Each domain in the assessment was judged as having a low, unclear, or high risk of bias. We defined high-quality articles as those having a low risk of bias in four or more domains. Disagreements between the reviewers were resolved by discussion.

### Data Analysis

The results of each trial were combined using standard meta-analytic methods to estimate an overall effect for acupuncture-related therapies plus CM vs. CM. Since all outcomes were continuous variables, weighted mean differences (WMDs), with 95% CIs, were calculated using RevMan 5.4.1 (Reviewer Manager Software 5.4.1; Cochrane Collaboration, Oxford, UK) (Fleiss, 1993). Statistical heterogeneity was assessed using the *I*^2^ statistic. When *P* ≥ 0.1 and *I*^2^ ≤ 50%, the included trials were considered to be homogeneous, and the fixed-effects model was used for analysis. However, when *P* < 0.1 and *I*^2^ > 50%, which implied that the included studies were heterogeneous, a random-effects model was used to obtain more reliable outcomes. For all analyses, *P* < 0.05 was considered statistically significant. For the primary outcomes, meaningful improvements were assessed by the minimal clinically important change (MCIC) (Schrag et al., 2006).

To investigate whether the treatment effect differed across the different intervention categories, we used subgroup analyses conducted for each primary and secondary outcome to perform subgroup difference tests. Subgroup analyses were performed for abdominal acupuncture, self-acupressure, catgut embedding, moxibustion, herbal decoction, and classical acupuncture treatment (AT). We use sensitivity analyses to judge the influence of each article on the overall effect of the results and to assess the robustness of our analysis. We removed each of the included studies one by one before the effect-size calculation and excluded articles that resulted in high heterogeneity or changed the pooled effect on outcomes.

Other methods of AT, such as BVA, acupoint injection, abdominal acupuncture, and auricular pressure, were analyzed in a qualitative manner because the number of included articles was too small or the data were too inadequate for extraction, and meta-analysis could not be carried out.

## Results

### Study Characteristics

**Figure 1:**
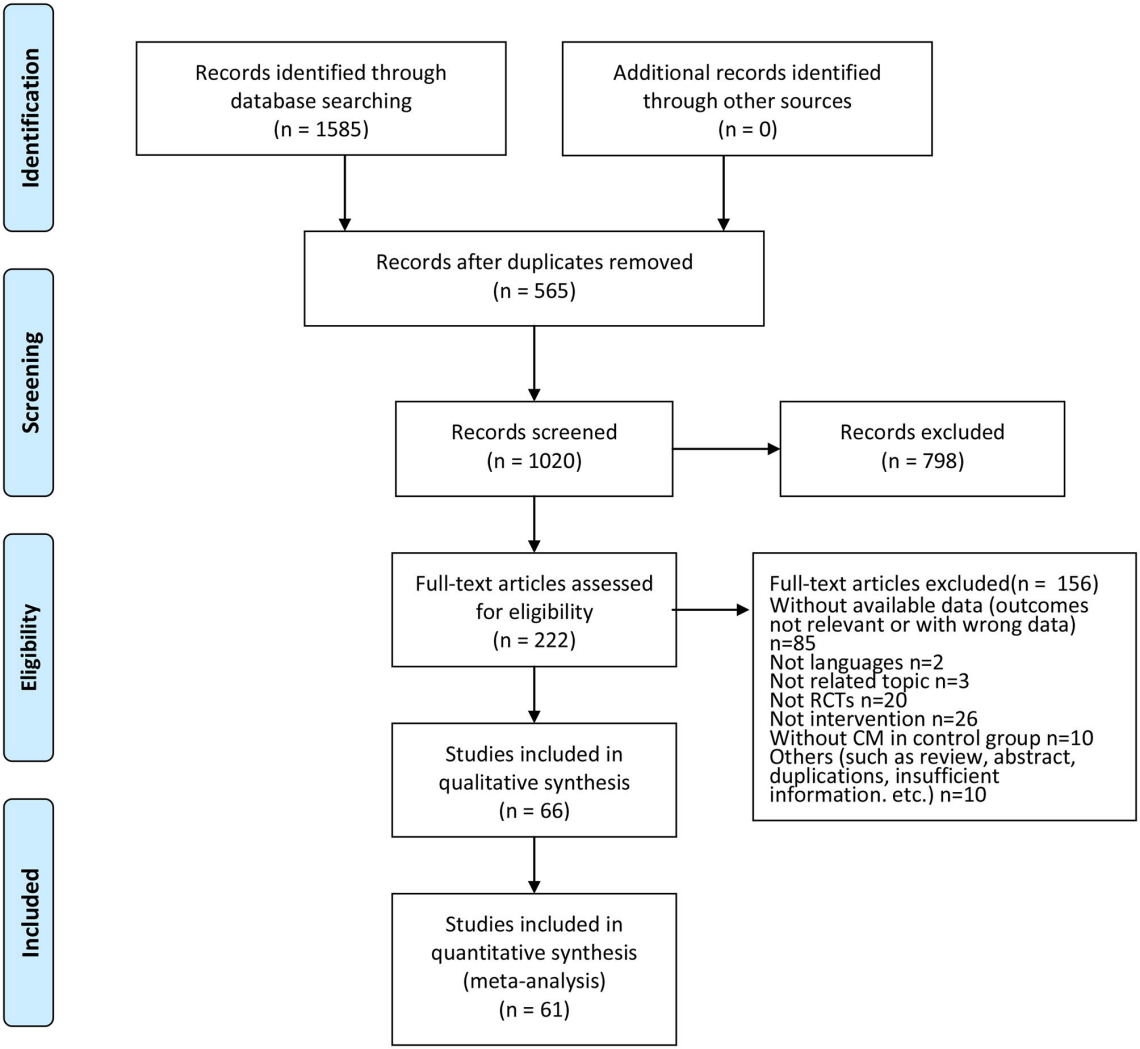
Flow of the trial selection process.

Initially, 1,585 potential articles were identified, of which 565 articles were excluded because of data duplication. After the titles and abstracts were screened, 798 of them were excluded. A further 156 articles were rejected due to lack of available data (the outcomes were not relevant or the data were inadequate), being in other languages, being on an unrelated topic, not being an RCT, not being an intervention, not including CM in the control group, or other reasons (such as a review, abstract, duplications, and insufficient information). The remaining 66 articles were read in full to establish eligibility. Of these 66 articles, five trials could not be meta-analyzed: (1) the data from one trial (Liu et al., 2015) could not be extracted because they were only available in graph form; (2) three trails (Wen et al., 2008; Hartmann et al., 2016; Cho S. Y. et al., 2018) published the changes in means and standard deviations; and (3) one trial (Cho et al., 2012) published only median and interquartile range data. Finally, 66 studies were included in this systematic review, and only 61 RCTs were included in the quantitative synthesis (Figure 1).

### Study Quality

**Figure 2:**
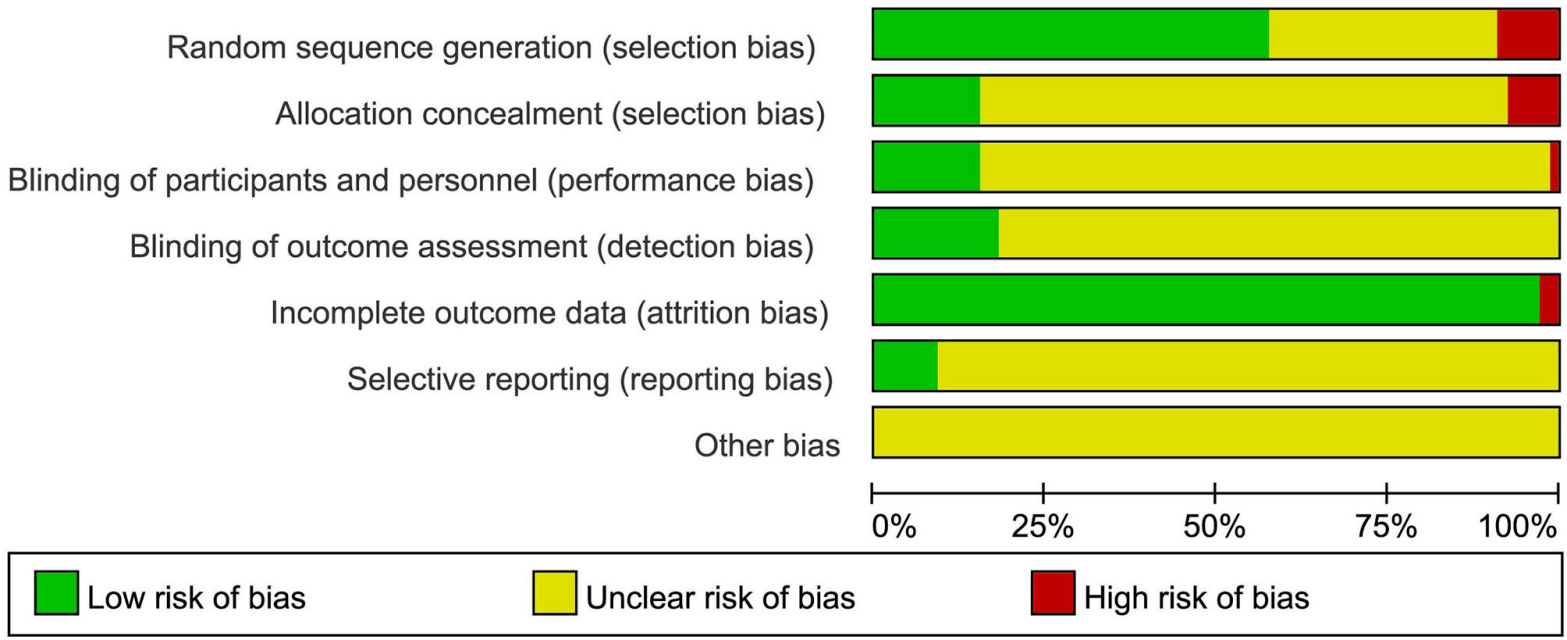
Demonstrate the risk of bias in the included studies.

**Figure 3:**
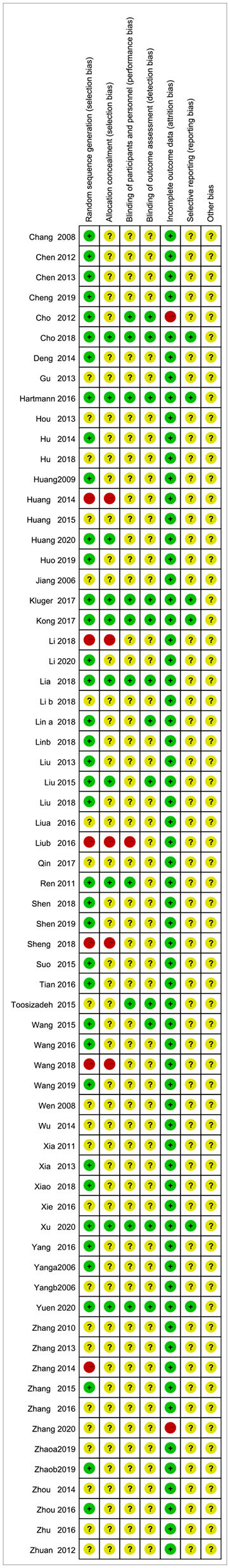
Risk of bias summary: review authors' judgements about each risk of bias item for each included study.

Thirty-nine (59.09%) studies described the methods of random sequence generation, 27 (40.91%) of which used a random number table, and seven (10.61%) used a computerized random number generator. Among these 39 studies, information on concealment of treatment allocation (using sealed opaque envelopes) was also poorly reported [5 (7.57%)]. Eleven (15.15%) studies showed a low risk of bias regarding the blinding of participants and personnel. Twelve (18.18%) studies were assessed as low risk regarding the blinding of assessors. Finally, only six (9.10%) studies reported protocols, and six (9.10%) studies stated intention to treat (ITT) as the primary method of analysis. Almost all the trials had no information on other risks of bias (Figures 2, 3).

### Interventions

The combination of acupuncture-related therapies with CM was adopted in the experimental group. Table 1 summarizes the detailed information on individual trials. Among them, 19 (28.79%) studies adopted special methods of AT: BVA (Cho et al., 2012; Hartmann et al., 2016; Cho S. Y. et al., 2018), abdominal acupuncture (Wen et al., 2008), acupoint injection (Liu et al., 2015), self-acupuncture (Yuen et al., 2020), catgut embedding (Hu et al., 2014; Zhu, 2016), moxibustion (Zhang et al., 2005; Deng et al., 2014; Wu, 2014; Shen et al., 2018, 2019), and herbal decoction (Zhang and Jiang, 2010; Hu, 2018; Xiao and Zhang, 2018; Cheng, 2019; Huo et al., 2019; Zhao, 2019).

**Table 1:** Characteristics of the included clinical trials involving acupuncture related therapy of Parkinson's Disease.

**References**	**Conducting country**	**Age (y)**	**Sex (F/M)**	**Disease duration (y)**	**Experiment group**	**Control group**	**Frequency (period)**	**Outcome measures**	**Results**	**Adverse events (group: number)**
**Bee venom acupuncture**										
Cho S. Y. et al. (2018)	Korea	A. 64.42 ± 8.24 B. 61.33 ± 8.20 C. 64.07 ± 6.33	A. 10/14 B. 16/8 C. 5 /10	A. 5.08 ± 3.68 B. 5.92 ± 4.18 C. 4.53 ± 3.25	A. AT+BVA B. SA +saline	C.CM	2 times a week for 12 weeks	1. UPDRSII+III 2. UPDRS II 3. UPDRS III 4. PIGD 5. PDQL 6. BDI 7. MXE 8. DCL	1.A>C, *P =* 0.001 A>B, *P =* 0.444 2.A>C, *P =* 0.001 A>B, *P =* 0.257 3. A>C, *P =* 0.008 A>B, *P =* 0.793 4.A>C, *P =* 0.001 A>B, *P =* 0.244	Mild pain or slight bleeding after AT; mild itchiness or mild swelling after BVA
Hartmann et al. (2016)	France	A. 60.3;15 B. 63.3;8 (Median; Interquartile range)	A. 12/8 B. 8/12	A. 5.9;4.4 B. 5.6;4 (Median; Interquartile range)	A. BV (100 ug)	B. Placebo (NaCl 0.9%, 1 mL)	Once a month over 11 months periods	1. UPDRSIII 2. BREF 3. MMS 4. LED 5. PDQ-39 scores 6. [123I]-FP-CIT	Not significant	Redness/itching at injection-site (B:6), insomnia (B:1, A:1), nausea (B:9, A:3), fatigue (B:10, A:2), dyskinesia (B:1, A:1), bradycardia (B:2, A:0)
Cho et al. (2012)	Korea	A. 55.0 (52.0, 66.0)B. 57.0 (49.0, 69.0)C. 57.0 (48.0, 68.0) (Median; Interquartile range)	A. 8/5 B. 8/5C. 6/3	A. 6.0 (3.0,9.0) B. 5.0 (2.0,10.0) C. 5.0 (4.0,7.0) (Median; Interquartile range)	A. AT+CM B. BVA+CM	C.Waiting group (CM)	Twice a week for 8 weeks	1. Total UPDRS (I–V) 2. UPDRSI 3. UPDRS II 4. UPDRS III 5. UPDRSIV 6. UPDRSV 7. UPDRSVI 8. BBS 9. 30-m walking time 10. steps to walk 30-m 11. PDQL 12. BDI	1.3.4 B>C, *P* < 0.05	Itchiness (B:1) (eliminated from the study). No other serious adverse events reported
**Acupoint injection**										
Liu et al. (2015)	China	A. 62.9 ± 8.4 B. 64.8 ± 8.1 C. 65.2 ± 9.8	A. 16/29 B. 19/26 C. 20/25	A. 6.4 ± 2.9 B. 5.8 ± 2.5 C. 6.0 ± 3.1	A. AT+ Madopar B. Acupoint injection of kakkonein+ Madopar	C.Madopar	3 times a week (only Monday/ Wednesday/ Friday) for 8 weeks	1. UPDRSI 2. UPDRSII 3. UPDRSIII	1. B>C, A>C, *P* < 0.05 A,B Not significant 2. B>A, B>C, A>C, *P < * 0.05	NR
**Abdominal acupuncture**										
Wen et al. (2008)	China	62.14 ± 9.25	23/37	NR	A. Abdominal AT+ Moxibustion *n* = 30	B. Madopar *n* = 30	Once daily, 3 times a week for 3 months	1. UPDRS Total (I–IV) 2. UPDRS 3. UPDRS II 4. UPDRS III 5. UPDRSIV	1.3 A>B, *P < * 0.05 2.4.5 Not significant	Ns
**Self-acupressure**										
Yuen et al. (2020)	China	A. 63.77 ± 1.41 B. 64.64 ± 2.57	A. 13/9 B. 10/4	A. 8.41 ± 1.27 B. 8.64 ± 1.44	A. conduction exercise and self-acupressure	B. No additional treatment	Once daily, twice at maximum for 8 weeks	1. CPDQ-39 total 2. CDQ total	Not significant	Leg bruising (A:1) Upper respiratory infection (A:1; B:1) Fall (A:1) Change of dosage of PD medication (A:1)
**Catgut embedding**										
Zhu (2016)	China	NR	NR	NR	A. acupoint catgut embedding +CM *n* = 31	B. CM *n* = 31	Every 15 days a time for 4 times	1. UPDRS	1.A>B, *P < * 0.05	NR
Hu et al. (2014)	China	A. 68.2 ± 1.2 B. 67.9 ± 5.4	A. 10/30 B. 11/29	A. 6.08 ± 9.5 B. 5.98 ± 6.36	A. Bushenzhichan Decoction+ catgut embedding+Madopar	B. Madopar	Decoction: once daily for 3months Catgut embedding Once daily or every 3 days for 3 months	1. UPDRSI 2. UPDRSII 3. UPDRSIII 4. UPDRS-Total 5. DosageofMadopar	1.2.3.4 A>B, *P < * 0.05	No abnormal changes in biochemical indices
**Moxibustion**										
Shen et al. (2019)	China	A. 73.9 ± 11.5 B. 71.9 ± 11.4	A. 15/21 B. 16/20	A. 8.5 ± 1.6 B. 7.1 ± 1.5	A. AT+Warming Needle Moxibustion+B	B. CM+hemp seed soft capsule	Every other day for 28 days	1. UPDRSII 2. UPDRSIII 3. constipation score 4. medication compliance	1.2 A>B, *P < * 0.05	NR
Deng et al. (2014)	China	A. 58 ± 6.0 B. 60 ± 5.7	A. 5/10 B. 6/9	A. 4.1 ± 1.5 B. 4.6 ± 1.2	A. ScalpEA+ Warming Needle Moxibustion+CM	B. CM (Madopar+ Pramipexole)	8 weeks	1. UPDRS-Total 2. total efficiency of UPDRS	1.A>B, *P < * 0.05 2.A>B, *P < * 0.01	NR
Shen et al. (2018)	China	A. 74 ± 11 B. 72 ± 11	A. 12/18 B. 13/17	A. 8.53 ± 1.55 B. 7.13 ± 1.47	A. AT+ Warming Needle Moxibustion+B	B. CM+hemp seed soft capsule	Every other day for 4 weeks	1. BBS 2. PAC-QOL 3. UPDRS III	1.2 A>B, *P < * 0.05	NR
Wu (2014)	China	69.3 ± 11.3	NR	3.57 ± 1.21	A. mild moxibustion+ Madopar *n* = 33	B. Madopar *n* = 32	Once daily for 15 days	1. UPDRSI 2. UPDRSII 3. UPDRSIII 4. UPDRSIV 5. UPDRS-Total 6. total efficiency of Webster score	2.6 A>B, *P < * 0.05	NR
Zhang et al. (2005)	China	51–78 (66.8)	25/29	2 years−9 years	A. moxibustion (herbs-artitioned) +CM *n* = 54	B. CM *n* = 36	Every other day for 15 days as a course for 2 courses, resting for 2–3days between 2 courses	1. total efficiency of UPDRS	1.A>B, *P < * 0.05	NR
**Herbal Decoction**										
Zhao (2019)	China	A. 55.76 ± 17.26 B. 57.54 ± 19.05	A. 28/32 B. 26/34	A. 5.87 ± 19.03 B. 5.37 ± 20.98	A. AT+Shuyuzhi chan Decoction + Madopar	B. Madopar+Fluoxetine	Once daily for 8 weeks	1. HAMD 2. UPDRS 3. DA	1.2.3 A>B, *P < * 0.05	NR
Huo et al. (2019)	China	A. 65.12 ± 8.25 B. 64.89 ± 8.17	A. 15/26 B. 17/24	A. 7.27 ± 1.81 B. 7.31 ± 1.76	A. AT+GuiluerxianDecoction+CM	B. CM (Madopar+Pramipexole) +Alprazolam	Once daily for 4 weeks	1. SL/TST/AT 2. Nrem-S1–S4 3. PDSS 4. HAMD 5. UPDRS III 6. TCM symptom grading 7.5-HT 8. SP	1–8 A>B, *P < * 0.05	NR
Cheng (2019)	China	A. 65.69 ± 5.21 B. 65.63 ± 5.23	A. 17/28 B. 16/29	A. 3.19 ± 1.22 B. 3.12 ± 1.23	A. ScalpAT+Shaogan Dingchan Decoction+CM	B. CM	Once every other day, 3 times a week for 3 months	1. UPDRS-Total 2. patient satisfaction 3. complication rate 4. recurrence rate in 1 year	1.A>B, *P < * 0.05	NR
Xiao and Zhang (2018)	China	A. 62.28 ± 7.43 B. 63.83 ± 7.62	A. 19/29 B. 21/27	A. 7.27 ± 3.24 B. 8.32 ± 3.57	A. EA+Huangqibu shen Decoction+CM	B. CM (Madopar)	Once daily for 2 months	1. hs-CRP/TNF-/IL-6 2. MMSE 3. ADL	1.A>B, *P < * 0.05 2.A>B, *P =* 0.013 3.A>B, *P =* 0.006	Chest distress (B:2) insomnia (B:1) anorexia (B:2) dizzy (A:1, B:2) drowsiness (A:1, B:2)
Hu (2018)	China	A. 66.60 ± 4.82 B. 66.42 ± 4.78	A. 18/37 B. 20/35	A. 14.70 ± 3.85 B. 14.62 ± 3.81(months)	A. AT+Bushentongqiao Decoction+ Carbidopa	B. Carbidopa	Once daily for 6 months	1. TCM syndrome integral 2. UPDRSI/II/III 3.P DQ-39 4.SAS 5. SDS 6.IL-1β 7.IL-6 8.SOD 9.MDA	1–9 A>B, *P < * 0.05	NR
Zhang and Jiang (2010)	China	A. 69.3 ± 7.6 B. 67.8 ± 9.1	A. 15/17 B. 18/14	A. 4.67 ± 2.10 B. 5.08 ± 1.32	A. Bushenhuoxue Decoction+AT+ B	B. Madopar	Once daily for 3 weeks as a course for 3 courses, resting a week between 2 courses	1. UPDRSII 2. UPDRSIII 3. UPDRSIV 4. UPDRS-Total 5. H-YStage 6. Dosageof Madopar 7. total efficiency of UPDRS	1.2.4 A>B, *P < * 0.05 3.Not significant	NR
**Acupuncture Treatment**										
Xu et al. (2020)	China	A. 61.7 ± 10.28 B. 61.95 ± 9.77	A. 18/15 B. 16/21	A. 3.52 ± 2.78 B. 3.26 ± 2.32	A. EA+Madopar	B. Madopar	4 days per week for 8 weeks	1. UPDRSI 2. UPDRS II 3. UPDRS III 4. UPDRSIV 5. Webster 6. PDSS 7. SDS	1.A>B, *P < * 0.05 2. A>B, *P =* 0.121 3. A>B, *P =* 0.054 4. A>B, *P < * 0.05 5. A>B, *P < * 0.05 6. A>B, *P =* 0.001 7. A>B, *P < * 0.05	No serious adverse events reported
Li Z. et al. (2018)	China	A. 65.79 ± 6.07 B. 62.85 ± 5.00 C. 62.17 ± 7.66	A. 6/8 B. 6/7 C. 3/9	A. 5.14 ± 3.32 B. 5.03 ± 4.73 C. 7.33 ± 4.62	A. AT+CM B. SA+CM	C.WG+CM	Twice weekly for 12 weeks	1. UPDRSII 2. UPDRS III 3. PIGD 4. fMRI: DC/ReHo/ALFF	NR	NR
Kong et al. (2017)	China	A. 66.4 ± 6.5 B. 62.9 ± 9.7	A. 14/6 B. 13/7	A. 87.2 ± 53.2 B. 50.1 ± 26.4 (months)	A. AT	B. SA	Twice weekly for 5 weeks	1. MFI-General fatigue 2. MFI-Total 3.UPDRSIII 4. PDQ-39 5. GDS 6. ESS	1.A>B, *P =* 0.09 2–6, Not significant	1.a skull fracture after a fall; 2. A pelvic fracture, also after a fall 3. exacerbation of anxiety
Kluger et al. (2016)	USA	A. 64.4 ± 10.3 B. 63.0 ± 13.0	A. 17/30 B. 18/29	NR	A. AT+CM	B. SA+CM	Twice-weekly sessions at least 1 day apart for 6 weeks	1. MFIS-Total 2. MFIS-Physical 3. MFIS-Cognitive 4. MFIS-Psychosocial 5. UPDRS III 6. PDQ-39 7. HADS Anxiety 8. HADS-Depression 9. PDSS 10. ESS 11. AES	Not significant	Constipation (A:1)
Wang et al. (2016)	China	A. 61 ± 10 B. 62 ± 9 C. 61 ± 8	A. 15/15 B. 14/16 C. 15/16	A. 5.63 ± 1.83 B. 5.57 ± 1.55 C. 6.12 ± 1.31	A. EA+Madopar (2/100 HZ) B. EA+Madopar (100 HZ)	C.Madopar	3 times per week for 90 days	1. UPDRS 2. TAS	1.B>C, *P < * 0.01 2.B>C, *P < * 0.01	NR
Xie et al. (2016)	China	71.51 ± 6. 06	47/61	5.7 ± 4. 1	A. AT+CM+Triple Viable Bacteria *n* = 53	B:CM+Triple Viable Bacteria *n* = 53	Once daily for 8 weeks	1. HAMD 2. UPDRSI 3. UPDRS II 4. UPDRSIII 5. UPDRSIV 6. UPDRS-Total	1.A>B, *P* < 0.05 6.A>B, *P* < 0.05	NR
Toosizadeh et al. (2015)	USA	A. 69.8 ± 4.5 B. 71.0 ± 11.7	A. 4/6 B. 3/2	A. 3.0 ± 1.0 B. 2.9 ± 0.7	A. EA (4 Hz or 100 Hz)	B. ShamEA (non-acupuncture points, just turning on the light of the stimulator)	Once a week for 3 weeks	1. GOG_AP_sway 2. GOG_ML_sway 3. GOG_ML/AP_sway 4. ankle sway 5. hip sway 6. ankle/hip.sway 7. SF-12(PCS) 8. SF-12(MCS) 9. ShortFES-I 10.VAS 11. UPDRS-Fall 12.UPDRS-Rigidity 13.UPDRSI 14.UPDRSII 15.UPDRS III	10. Not significant11. A>B, *P* = 0.391 2.A>B, *P* =0.051 3. A>B, *P < * 0.011 4. A>B, *P* =0.021 5.A>B, *P < * 0.001	NR
Wang et al. (2015)	China	A. 62.1 ± 8.7 B. 59.1 ± 12.4	A. 15/13 B. 11/9	A. 2.9 ± 2.9 B. 2.7 ± 2.3	A. EA+CM (anti-Parkinsonian drugs)	B. CM (anti-Parkinsonian drugs)	Once every 3 days for 2 months	1. UPDRSII 2. UPDRSIII 3. UPDRSIV 4. H-Y Stage 5. MoCA 8. HAMD 9. PSQI 10. ADL 11. PDQ-39 12. NO/TNF-α/IL-1β/PGE2 13. DA/Ach/NE/5-HT	2.A>B, *P =* 0.036 4.5.6.7.8. Not significant 9.A>B, *P =* 0.034	NR
Chen et al. (2012)	China	A. 65.60 ± 3.79 B. 61.93 ± 3.67	A. 11/19 B. 13/17	A. 5.4 ± 1.75 B. 6.4 ± 2.15	A. EA+B	B. medication (MadoparandTolterodine)	6 times a week (except Sunday) for 6 weeks	1. frequency of average urination of 24 h 2. frequency of incontinence of 24 h 3. average urine volume at a time 4. UPDRSIII	1.2.3.4 A>B, *P < * 0.05	Dry mouth, dry eye, blurred vision, decreased reaction/ intelligence/activity (A:3, B:12), Urinary retention (B:1), ALT, AST mild elevation (B:1)
Yang et al. (2006a)	China	49–73 (63)	14/24 (Total)	8 months−6 years 1 month	A. ScalpET+AT +B *n* = 19	B. medication (Benserazide-Levodopa) *n* = 19	Every other day for 10 times as 1 course for 4 courses, resting for 7 days between 2 courses	1. UPDRS 2. SODandcontene of peroxidation lipid	1.2 A>B, *P < * 0.05	NR
Jiang X. M. et al. (2006)	China	A. 65.60 ± 3.78 B. 60.80 ± 3.63	A. 7/8 B. 9/6	A. 5.40 ± 1.75 B. 6.4 ± 2.14	A. Scalp EA +Madopar	B. Madopar	5 times 1 week for 6 weeks (total 30 times)	1.Webster 2. UPDRSIII	1.Not significant 2.A>B, *P < * 0.05	Pain on acupuncture area (A:1), Digestive disorders (B:5), On-off phenomenon (B:1)
Chang et al. (2008)	China	A. 58.2 ± 12.3 B. 57.6 ± 11.9	A. 12/18 B. 11/19	A. 3.4 ± 1.3 B. 3.6 ± 1.5	A. AT+EA+ Madopar	B. Madopar	Once daily for 30 days	1. UPDRS	1.A>B, *P < * 0.05	NR
Zhang et al. (2020)	China	A. 52.16 ± 3.56 B. 55.32 ± 3.02	A. 27/21 B. 22/26	A. 4.46 ± 1.32 B. 4.12 ± 1.21	A. EA+ Pramipexole	B. Pramipexole	6 times a week for 8 weeks	1. UPDRS 2. HAMD 3. PDSS 4. YKL-40 5. BDNF	1.2.3.4.5 A>B, *P < * 0.05	NR
Huang et al. (2020)	China	A. 62.25 ± 9.31 B. 61.94 ± 8.41	A. 28/52 B. 30/49	A. 3.71 ± 0.77 B. 3.59 ± 0.65	A. AT+ medication	B. medication (Madopar+Bazhen Decoction and Tianmagouteng Decoction)	6 times a week for 8 weeks	1. dysfunction of tremor syndrome 2. PDQ-39 SI 3. symptoms of syndrome of Qi and blood deficiency 4. UPDRSI-III 5. NMSQuest 6. NMSS	1–5 A>B, *P < * 0.01	NR
Zhao and Wang (2019)	China	A. 66.63 ± 5.71 B. 64.85 ± 6.14	A. 15/17 B. 15/16	A. 4.5 ± 2.7 B. 4.2 ± 2.9	A. AT+B	B. CM+ Paroxetine	6 times a week for 4 weeks	1. HAMD 2. UPDRS	1.A>B, *P =* 0.000 2.A>B, *P =* 0.019	NR
Li et al. (2020)	China	A. 71 ± 5 B. 68 ± 7	A. 16/14 B. 17/13	A. 6.2(6.49,9.16) B. 6.00(6.67,9.57)	A. ScalpEA+AT+CM	B. CM	3 times a week for 12 weeks	1. UPDRS 2.20-m walking time and average interval 3. PDQ-39	1,3 A>B, *P < * 0.05	NR
Wang et al. (2019)	China	A. 66.24 ± 6.09 B. 65.72 ± 5.67 C. 65.43 ± 6.82	A. 18/14 B. 17/15 C. 15/16	A. 6.55 ± 3.32 B. 7.15 ± 3.47 C. 6.82 ± 2.98	A. AT+ Madopar B. AT	C. Madopar	Once daily for 30 days	1. UPDRSI 2. UPDRSII 3. UPDRSIII 4. UPDRSIV 5. UPDRS-Total 6. MoCA 7. MMSE 8. ADL	1. A>C, *P < * 0.01 A>B, *P < * 0.05, B>C, *P < * 0.05 2.3.4.5 A>C, *P < * 0.05, A>B, *P < * 0.05 5. B>C, *P < * 0.05 6.7. A>C, *P < * 0.05, 8. A>C, *P < * 0.01, A>B, *P < * 0.05	NR
Li and Wang (2018)	China	A. 72.16 ± 5.41 B. 73.36 ± 6.18	A. 11/19 B. 16/14	A. 5.62 ± 2.31 B. 5.14 ± 3.14 (months)	A. AT+ Madopar	B Madopar	Once daily for 6 weeks	1. MoCA 2. MMSE	1.2 A>B, *P < * 0.05	NR
Lin Z. C. et al. (2018)	China	A. 58.3 ± 4.6 B. 59.2 ± 4.4	A. 18 B. 17	A. 3.3 ± 0.9 B. 3.2 ± 1.0	A. ScalpEA+B	B. Donepezil + Cognitive training+CM	6 times a week for 6 weeks	1. TMT-A/B 2. MOCA 3. UPDRSII 4. UPDRSIII	1.2.3.4 A>B, *P < * 0.05	Dizzy (A:1, B:1) tremor progress (A:1) stomach upset (B:1)
Wang et al. (2018)	China	A. 56.0 ± 5.6 B. 55.0 ± 6.1	A. 10/20 B. 8/22	A. 7.0 ± 1.5 B. 8.0 ± 2.0	A. ScalpEA+CM	B. CM (Madopar)	5 times a week for 6 weeks	1. UPDRS	1. A>B, *P < * 0.05	NR
Li G. S. et al. (2018)	China	A. 53.64 ± 7.18 B. 52.98 ± 7.52	A. 20/19 B. 21/18	A. 2.10 ± 1.34 B. 2.08 ± 1.52	A. EA+Madopar	B. Madopar	3 times a week for 12 weeks	1. UPDRS	1. A>B, *P < * 0.05	NR
Lin D. et al. (2018)	China	A. 63.4 ± 4.3 B. 61.6 ± 5.8	A. 18/12 B. 20/10	A. 5.3 ± 2.6 B. 4.7 ± 2.3	A. AT+CM	B. CM	Once daily for 4 weeks	1. UPDRSI 2. UPDRSII 3. UPDRSIII	1.2.3. Not significant 4.5.6.A>B, *P < * 0.05	NR
								4. UPDRSIV 5. UPDRS-Total 6. WOQ-9		
Liu et al. (2018)	China	A. 64.68 ± 7.32 B. 63.78 ± 7.51	A. 24/29 B. 26/27	A. 5.87 ± 2.54 B. 5.66 ± 2.78	A. EA+AT+ Carbidopa	B. Carbidopa	Once daily for 3 months	1. MDA 2.SOD 3.CystainC 4. UPDRS 5. PDQ-39	1–5 A>B, *P < * 0.05	NR
Sheng and Guo (2018)	China	A. 60.28 ± 3.15 B. 61.07 ± 3.28	A. 18/20 B. 19/19	NR	A. AT+ Madopar	B. Madopar	Once daily for 15 days as 1 course for 3 courses, resting for 5–7 days between 2 courses	1. dosage of Madopar 2. clinical effects 3. assessmentsresultsof motor function	1.2.3 A>B, *P < * 0.05	NR
Qin et al. (2016)	China	A. 45-79 B. 46-65	A. 15/30 B. 14/16	NR	A. EA+ Madopar	B. Madopar	Once daily for 20 days	1. dosageofMadopar	1. A>B, *P < * 0.05	NR
Tian et al. (2016)	China	A. 62.4 ± 5.1 B. 60.8 ± 4.4	A. 9/41 B. 12/38	A. 3.3 ± 0.5 B. 3.6 ± 0.4	A. EA+B	B. Madopar+Fluoxetine	Every other day for 3 months	1. HAMD 2. BDNF	1.2. A>B, *P < * 0.05	NR
Yang (2016)	China	A. 73.45 ± 7.21 B. 76.86 ± 8.24	A. 6/14 B. 9/11	NR	A. EA+ levodopa	B. levodopa	Once daily for 30 days	1. UPDRS 2. Webster	1. A>B, *P < * 0.05 2. Not significant	NR
Zhang (2016)	China	A. 58.4 ± 6.9 B. 54.6 ± 7.6	A. 16/24 B. 18/22	A. 28.3 ± 10.2 B. 26.4 ± 12.8(months)	A. AT+ Madopar	B. Madopar	Once daily for 45 days	1. UPDRS	1. A>B, *P < * 0.05	NR
Zhou et al. (2016)	China	A. 69.2 ± 4.6 B. 70.1 ± 4.4	A. 33/30 B. 32/32	A. 7.43 ± 3.85 B. 9.32 ± 3.45	A. Scalp EA+ Madopar	B. Madopar	Once daily for 8 weeks	1. UPDRSII+III 2. PDSS	1.2 A>B, *P < * 0.05	Dizzy (A:2) epigastric discomfort (A:1) drowsiness, (A:1) hypotension (A:1, B:3) insomnia (B:3) chest distress (B:3) constipation (B:2) paropsia (B:1) anorexia (B1)
Liu B. (2016)	China	A. 65.65 ± 4.15 B. 65.59 ± 4.18	A. 18/21 B. 16/19	A. 4.41 ± 2.01 B. 4.33 ± 2.04	A. EA+ Madopar	B. Madopar	3 times a week for 12 weeks	1. UPDRSI 2. UPDRSII 3. UPDRSIII 4. UPDRSIV	1.2.3.4 A>B, *P < * 0.05	NR
Liu and Wu (2016)	China	A. 59 ± 8 B. 63 ± 4	A. 11/9 B. 8/12	A. 3.4 ± 3.2 B. 4.1 ± 2.7	A. ScalpEA+ Madopar	B. Madopar	3 times a week for 30 times	1. frequency of static tremor (EMG) 2. UPDRSIII	1.2 A>B, *P < * 0.05	NR
Suo et al. (2015)	China	A. 67.2 ± 5.6 B. 66.8 ± 5.8	A. 12/23 B. 10/25	NR	A. Neck Points plus ScalpeEA+ Madopar	B. Madopar	6 times a week for 4 weeks	1. UPDRS 2. total efficiency of Webster	1.2. A>B, *P < * 0.05	NR
Huang et al. (2015)	China	62.7 ± 2.5	32/88	NR	A. Transcranial repetitiveAT (Dance tremor area)+ Madopar *n* = 40 B. AT (Dance tremor area)+ Madopar *n* = 40	C.Madopar *n* = 40	Once daily for 8 weeks	1. UPDRS	NR	NR
Zhang (2015)	China	A. 63 ± 9 B. 66 ± 6	A. 12/20 B. 10/18	A. 26.00 ± 11.97 B. 27.86 ± 9.59 (months)	A. AT+ Madopar	B. Madopar	Once daily for 30 days	1. UPDRS 2. total efficiency of UPDRS	1.2 A>B, *P < * 0.05	**NR**
Huang et al. (2014)	China	A. 61 ± 8 B. 59 ± 9	A. 7/13 B. 8/12	A. 34 ± 8 B. 35 ± 6	A. Scalp AT+ Madopar	B. Madopar	5 times a week for 4 weeks	1. UPDRS 2. PSQI	1. A>B, *P < * 0.001 2. A>B, *P < * 0.005	NR
Zhou et al. (2014)	China	A. 63.1 ± 4.54 B. 60.7 ± 3.19	A. 14/16 B. 13/17	A. 4.73 ± 1.68 B. 5.13 ± 2.04	A. AT+ Madopar	B. Madopar	Once daily for 10 days as 1 course for 4 courses, resting for 3 days between 2 courses	1. UPDRSI 2. UPDRSII 3. UPDRSIII 4. UPDRSIV	1.2.3. A>B, *P < * 0.05	NR
Gu et al. (2013)	China	A. 66 ± 8 B. 70 ± 8	A. 13/10 B. 10/15	A. 4.44 ± 3.32 B. 4.56 ± 3.11	A. EA+ Madopar	B. Madopar	3 times a week for 12 weeks	1. UPDRSI 2. UPDRSII 3. UPDRSIII 4. UPDRSIV 5. UPDRS-Total	1.2.4.5 A>B, *P < * 0.05 3. Not significant	NR
Xia et al. (2013)	China	A. 71.84 ± 7.22 B. 71.93 ± 8.12	A. 8/22 B. 10/20	A. 6.7 ± 3.0 B. 5.8 ± 4.0	A. EA+ Madopar+Fluoxetine	B. Madopar+Fluoxetine	Every other day for 3 months	1. HAMD 2. DA 3. total efficiency of HAMD	1.2.3. A>B, *P < * 0.05	NR
Chen et al. (2013)	China	A. 57–71 (62.00) B. 55–75 (65.05)	A. 14/16 B. 14/17	A. 4.8 B. 4.6 (average)	A. AT+ Madopar	B. Madopar	Every other day for 3 months	1. UPDRS 2. dosageof Madopar 3. total efficiency of UPDRS	1.3. A>B, *P < * 0.05 2. Not significant	No abnormal changes in biochemical indices
Liu and Jiang (2013)	China	A. 52 ± 8 B. 51 ± 6	A. 10/12 B. 11/10	A. 18.86 ± 7.95 B. 19.76 ± 6.93 (months)	A. AT+ Madopar	B. Madopar	Once daily for 30 days	1. UPDRS 2. H-Y stage	1.2 A>B, *P < * 0.05	NR
Hou et al. (2013)	China	A. 67 ± 10 B. 67 ± 8	A. 14/16 B. 12/18	A. 5.17 ± 1.84 B. 5.40 ± 1.75	A. EA+ Madopar	B. Madopar	3 times a week for 3 months	1. UPDRS 2. TAS	1.2 A>B, *P < * 0.01/0.05	NR
Zhuang and Zhuang (2012)	China	A. 61.27 ± 4.58 B. 61.21 ± 4.48	A. 12/19 B. 14/17	A. 5.28 ± 3.44 B. 4.98 ± 2.86	A. AT+ Madopar	B. Madopar	Once daily for 5 days as 1 course for 8 weeks, resting for 2 days between 2 courses	1. UPDRSI 2. UPDRSII 3. UPDRSIII 4. UPDRSIV 5. UPDRS-Total	1.2,3,4,5 A>B, *P < * 0.05	NR
Xia et al. (2011)	China	A. 71.94 ± 8.81 B. 71.33 ± 7.21	A. 8/14 B. 3/15	NR	A. EA+ Madopar	B. Madopar	Once daily for 20 days	1. UPDRS 2. Webster 3. total efficiency of Webster	1. Not significant 2. A>B, *P < * 0.05	NR
Ren et al. (2011)	China	A. 59.1 ± 12.1 B. 58.2 ± 11.9	A. 38/52 B. 41/49	A. 1.8 ± 0.3 B. 1.9 ± 0.4	A. AT+ Madopar	B. Madopar	Once daily for 30 days	1. UPDRSI 2. UPDRSII 3. UPDRSIII 4. UPDRSIV 5. UPDRS-Total 6. total efficiency of UPDRS	2.3.4.5.6 A>B, *P < * 0.05	NR
Huang et al. (2009)	China	A. 65.60 ± 3.78 B. 60.80 ± 3.63	A. 7/8 B. 9/6	A. 5.40 ± 1.75 B. 6.4 ± 2.14	A. Scalp EA+ Madopar	B. Madopar	Once daily for 6 times a week, for 5 weeks	1. UPDRSIII 2. SPECT-values of rCBF	1. A>B, *P < * 0.05	NR*Continue to next page*
Yang et al. (2006b)	China	49–73 (63.2)	11/15	8 months−5 years 7 months	A. ScalpEA+AT+ Madopar *n* = 13	B. Madopar *n* = 13	Once daily for 10 days as a course for 4 courses, resting 7 days between 2 courses	1. UPDRS 2. TCM syndrome integral 3. total efficiency of UPDRS	3. A>B, *P < * 0.05	Mild drymouth Nausea, and dizziness (A:1), mild dizziness (B:2), nausea (B:3), subcutaneous hematocele (B:2)
Zhang et al. (2013)	China	A. 66.80 ± 9.73 B. 66.67 ± 8.32	A. 14/17 B. 15/15	A. 8.92 ± 2.29 B. 7.79 ± 2.15	A. AT (Thick needle+ Madopar	B. Madopar	3 times a week for 3 months	1. UPDRS 2. TAS	1.2 A>B, *P < * 0.01	NR

*Ach, Acetylcholine; AES, Apathy Evaluation Scale; ALT, Alanine aminotransferase; AST, Aspartate aminotransferase; ALFF, Amplitudes of low-frequency fluctuation; AT, Acupuncture Treatment; BVA, bee venom acupuncture; BV, bee venom; BBS, Berg balance scale; BSS, The Bristol Stool Scale; BI, Barthel index; BDI, Beck Depression Inventory; CM, Conventional medication; COGAP sway, anterior-posterior central of gravity sway; COGML sway, medial-lateral central of gravity sway; COGML/AP sway, medial-lateral central of gravity sway to anterior-posterior sway; CDQ, custom-designed questionnaire; DA, dopamine; DCL, directional control; DC, degree centrality; EA, Electro-acupuncture; ESS, Epworth Sleepiness Scale; fMRI, functional Magnetic resonance imaging; FES-I, Short Falls Efficacy Scale-International; HADS, Hospital Anxiety and Depression Scale; HAMA, Hamilton Anxiety Scale; HAMD, Hamilton Depression Scale; H and Y, Hoehn and Yahr; IL-1β, the levels of interleukin-1β;IL-6, interleukin-6; MDA, malondialdehyde; MMSE, Mini-mental status examination; MoCA, Montreal cognitive assessment; MFI, the Multidimensional Fatigue Inventory; MFIS, Modified Fatigue Impact Scale; MXE, maximum excursion; NO, nitric oxide; NE, norepinephrine; NR, not reported; PAC-QOL, Patient Assessment of Constipation Quality of Life scale; PDQL, Parkinson's Disease Quality of Life Questionnaire; PDQ-39, 39-item Parkinson Disease Questionnaire; PDSS, Parkinson's Disease Sleep Scale; PIGD, postural instability and gait disturbance; PSG, polysomnography; PSQI, Pittsburgh Sleep Quality Index; PGE2, prostaglandinE2; ReHo, regional homogeneity; SA, sham acupuncture; SAG, sham acupuncture group; SAS, self-rating Anxiety Scale.; SD, standard deviation; SDS, self-rating Depression Scale; SF-12 (MCS), mental component summary of the SF-12 health survey; SF-12 (PCS), Physical component summary of the SF-12 health survey; SOD, superoxide dismutase; SP, substance P; TAG, true acupuncture group; TAS, Tension Assessment Scale; TCM, Traditional Chinese medicine; TNF- α, tumor necrosis factor- α; UPDRS, Unified Parkinson's Disease Rating Scale; UPDRS I, behavior and mood subscale of the Unified Parkinson's Disease Rating Scale; UPDRS II, activities of daily living subscale of the Unified Parkinson's Disease Rating Scale; UPDRS III, motor symptoms caused by Parkinson's Disease subscale of the Unified Parkinson's Disease Rating Scale; UPDRS IV, complications of therapy subscale of the Unified Parkinson's Disease Rating Scale; VAS, visual analog scale; 5-HT, 5-hydroxytryptamine; WG, waiting group; WHOQOL-BREF, Brief table of quality of Life measurement Scale (WHO);WOQ, The incidence rate of Wearing-off Questionnaire*.

*Studies denoted as a or b distinguishes those published by the same first author and in the same year.

The control group all received CM, while seven (10.61%) of them used additional drugs to address the complications of PD. Fluoxetine (three articles) (Xia et al., 2013; Tian et al., 2016; Zhao, 2019) and triple viable bacteria (one article) (Xie et al., 2016) were added to CM for PD-related depression. Tolterodine was used in one study (Chen et al., 2012) for PD-related overactive bladder syndrome. Alprazolam (Huo et al., 2019) and donepezil (Lin Z. C. et al., 2018) were used separately for PD-related sleepiness and cognitive decline.

### Effects of the Intervention

#### Primary Outcomes

##### Unified Parkinson's Disease Rating Scale-Total

**Figure 4:**
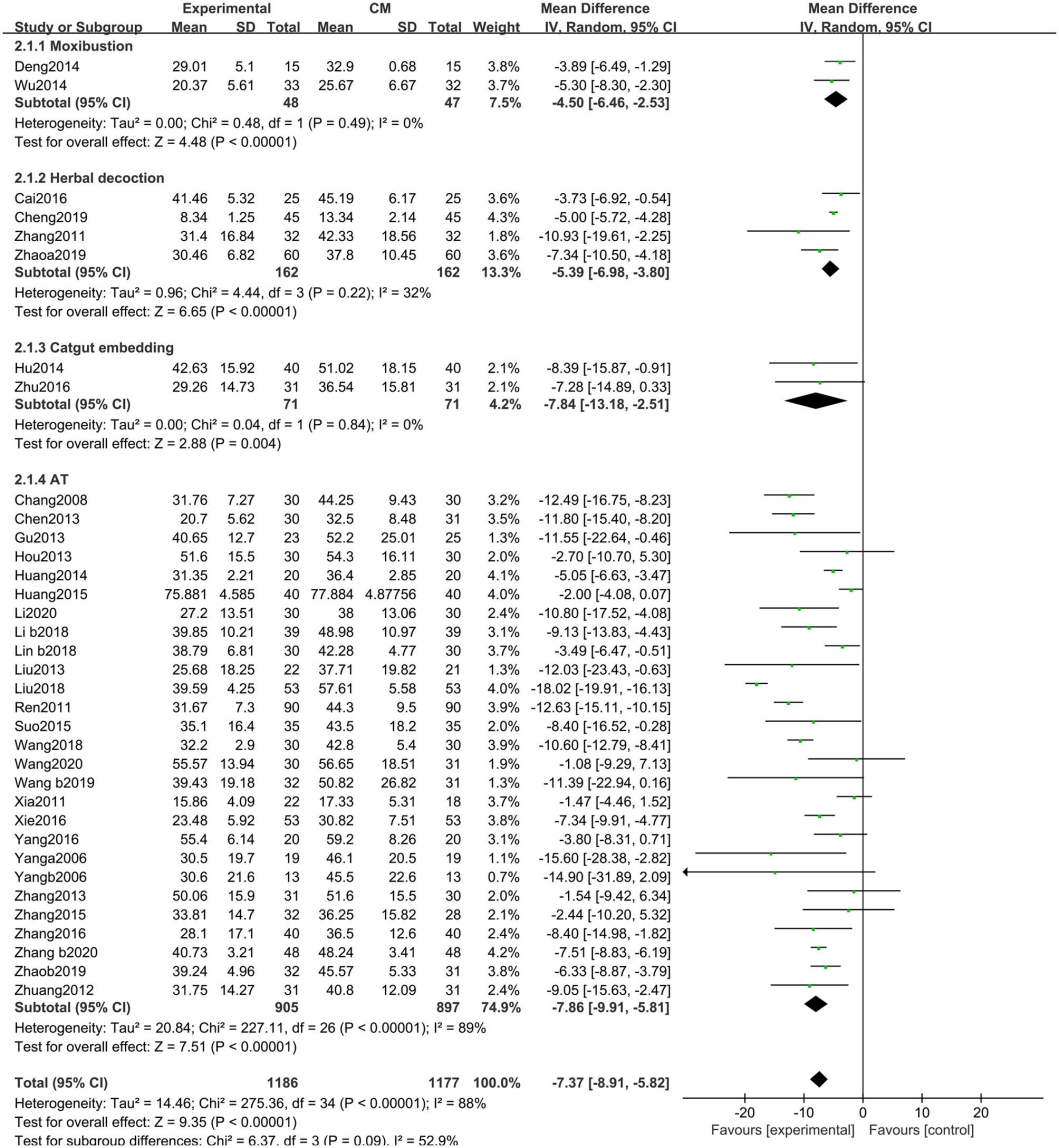
Comparison of acupuncture related therapies in terms of UPDRS-Total.

There were significant benefits on the UPDRS-total scores in the experimental group compared with the control group (MD: −7.37, 95% CIs: −8.91 to −5.82, *P* < 0.01). Heterogeneity existed across the trials (χ^2^ = 275.36, *P* < 0.01, *I*^2^ = 88%). Similarly, non-significant differences were observed among the subgroups (χ^2^ = 6.37, *P* = 0.09, *I*^2^ = 52.9%) (Figure 4).

##### Unified Parkinson's Disease Rating Scale-II (Activities of Daily Living)

**Figure 5:**
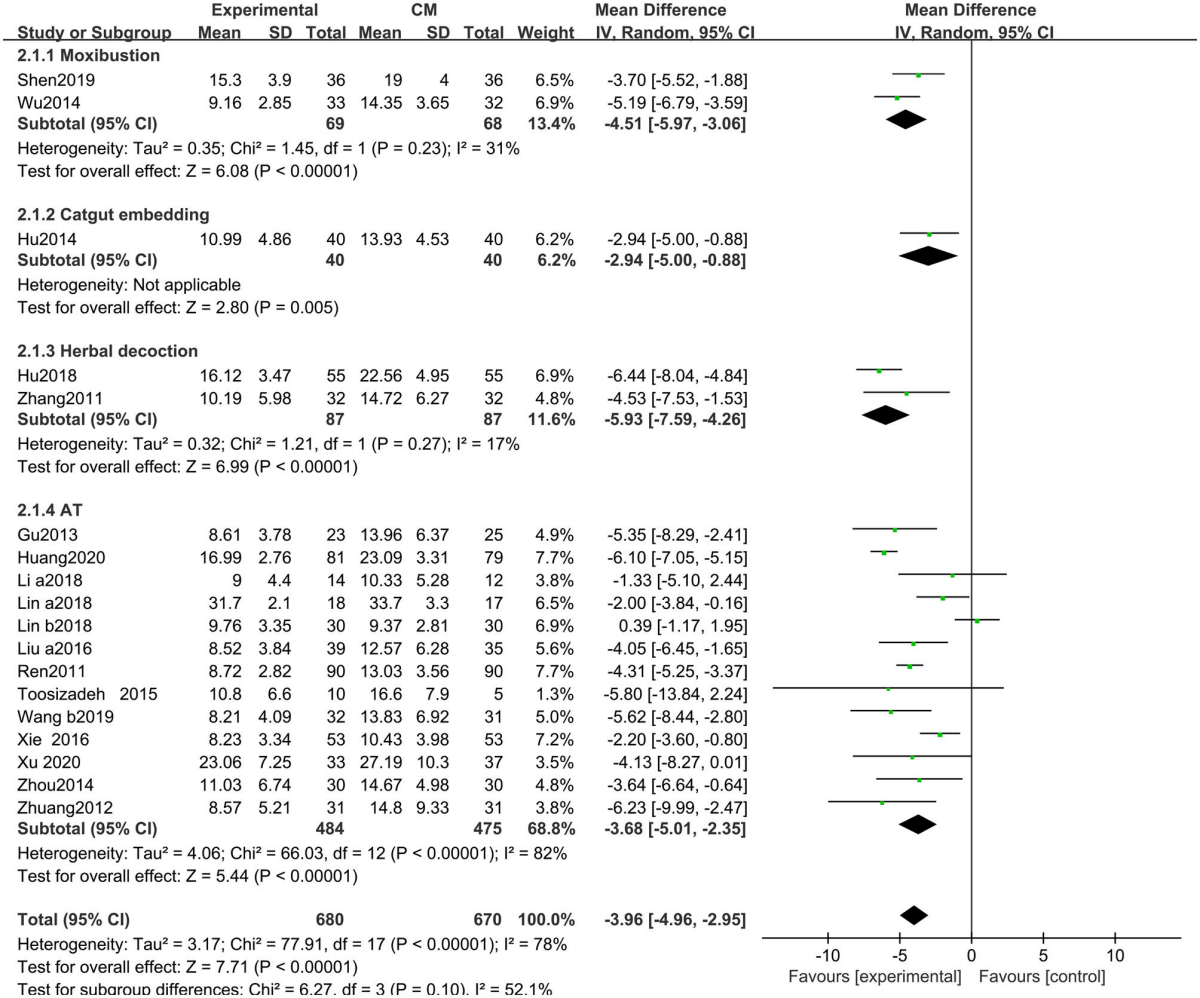
Comparison of acupuncture related therapies in terms of UPDRS-II.

The UPDRS-II scores improved with acupuncture-related therapies combined with CM (MD: −3.96, 95% CIs: −4.96 to −2.95; *P* < 0.01). Heterogeneity existed across the trials (χ^2^ = 77.91, *P* < 0.01, *I*^2^ = 78%). Similarly, non-significant differences were observed among the subgroups (χ^2^ = 6.27, *P* = 0.10, *I*^2^ = 52.1%) (Figure 5).

##### Unified Parkinson's Disease Rating Scale-III (Motor)

**Figure 6:**
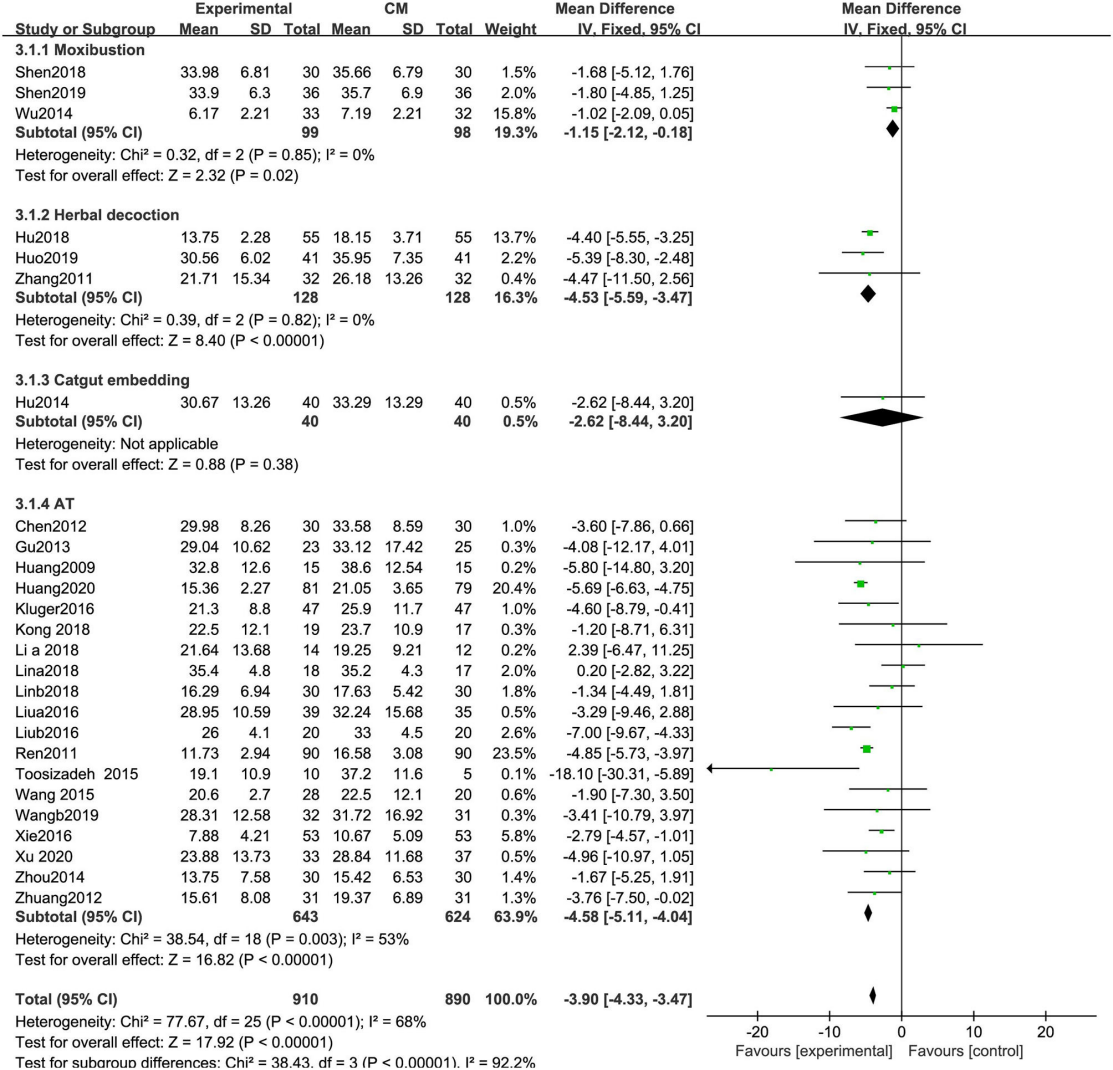
Comparison of acupuncture related therapies in terms of UPDRS-III.

UPDRS-III scores were significantly decreased in the experimental group compared with the control group (MD: −3.90, 95% CIs: −4.33 to −3.47, *P* < 0.01). Heterogeneity existed among the included trials (χ^2^ = 77.67, *P* < 0.01, *I*^2^ = 68%). Significant differences in UPDRS-III scores were observed among the subgroups of different types of acupuncture (χ^2^ = 38.43, *P* < 0.01; *I*^2^ = 92.2%) (Figure 6).

#### Secondary Outcomes

##### Unified Parkinson's Disease Rating Scale-I (Mentation, Behavior, and Mood)

**Figure 7:**
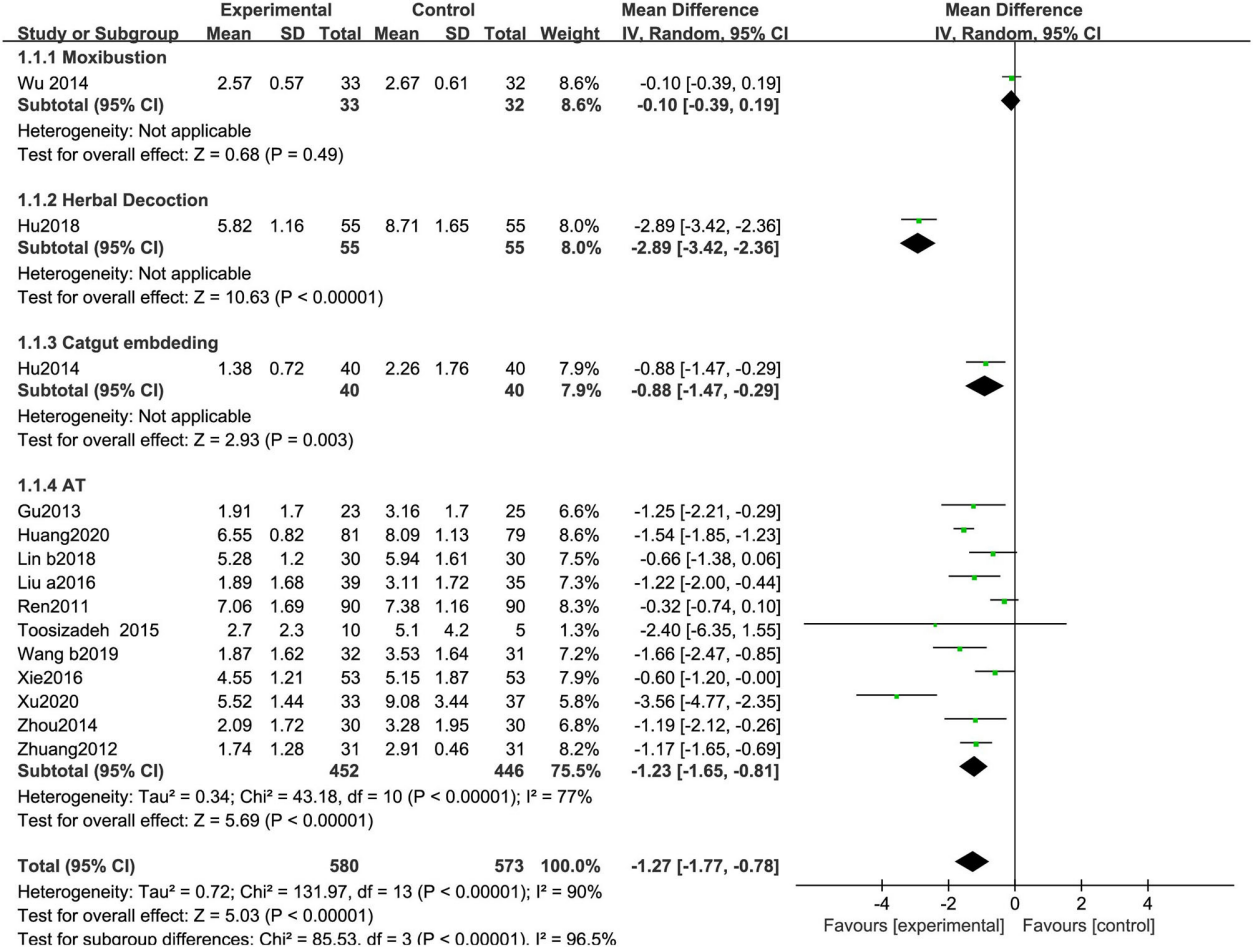
Comparison of acupuncture related therapies in terms of UPDRS-I.

As shown in Figure 7, 14 RCTs reporting UPDRS-I scores included 580 participants treated with acupuncture-related therapies and 573 participants treated with CM. The pooled results show a statistically significant improvement (MD: −1.27, 95% CI: −1.77 to −0.78, *P* < 0.01). The heterogeneity was significant (χ^2^ = 131.97, *P* < 0.01; *I*^2^ = 90%). Significant differences were observed among the subgroups (χ^2^ = 85.53, *P* < 0.01, *I*^2^ = 96.5%) (Figure 7).

##### Unified Parkinson's Disease Rating Scale-IV (Complications)

**Figure 8:**
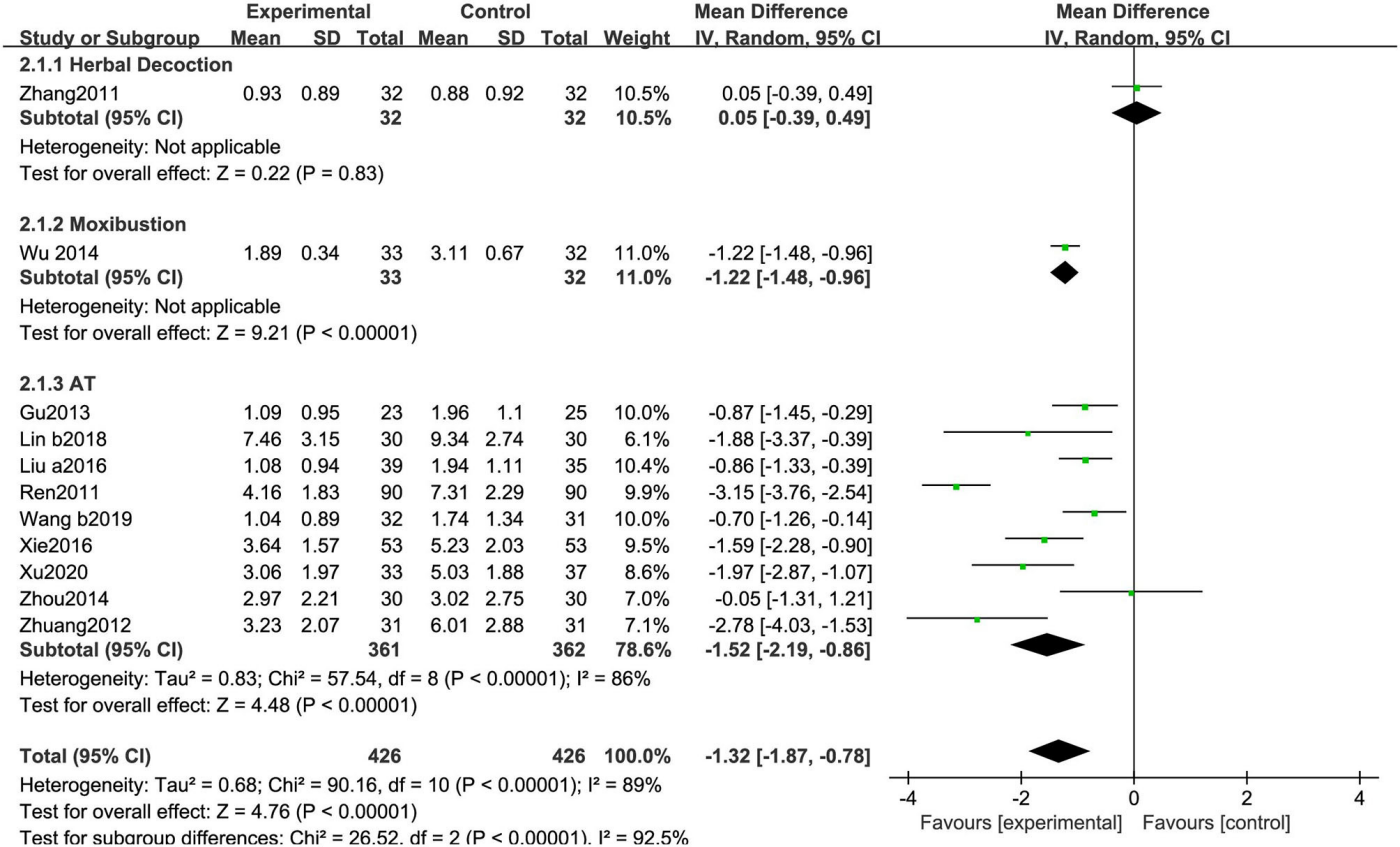
Comparison of acupuncture related therapies in terms of UPDRS-IV.

As shown in Figure 8, 11 RCTs using the UPDRS-IV included 852 participants. The meta-analysis showed that the experimental group was significantly superior to the CM group (MD: −1.32, 95% CI: −1.87 to −0.78, *P* < 0.01). The heterogeneity was significant (χ^2^ = 90.16, *P* < 0.01, *I*^2^ = 89%). Similarly, significant differences were observed among the subgroups (χ^2^ = 26.52, *P* < 0.01, *I*^2^ = 92.5%) (Figure 8).

##### 39-Item Parkinson's Disease Questionnaire

**Figure 9:**
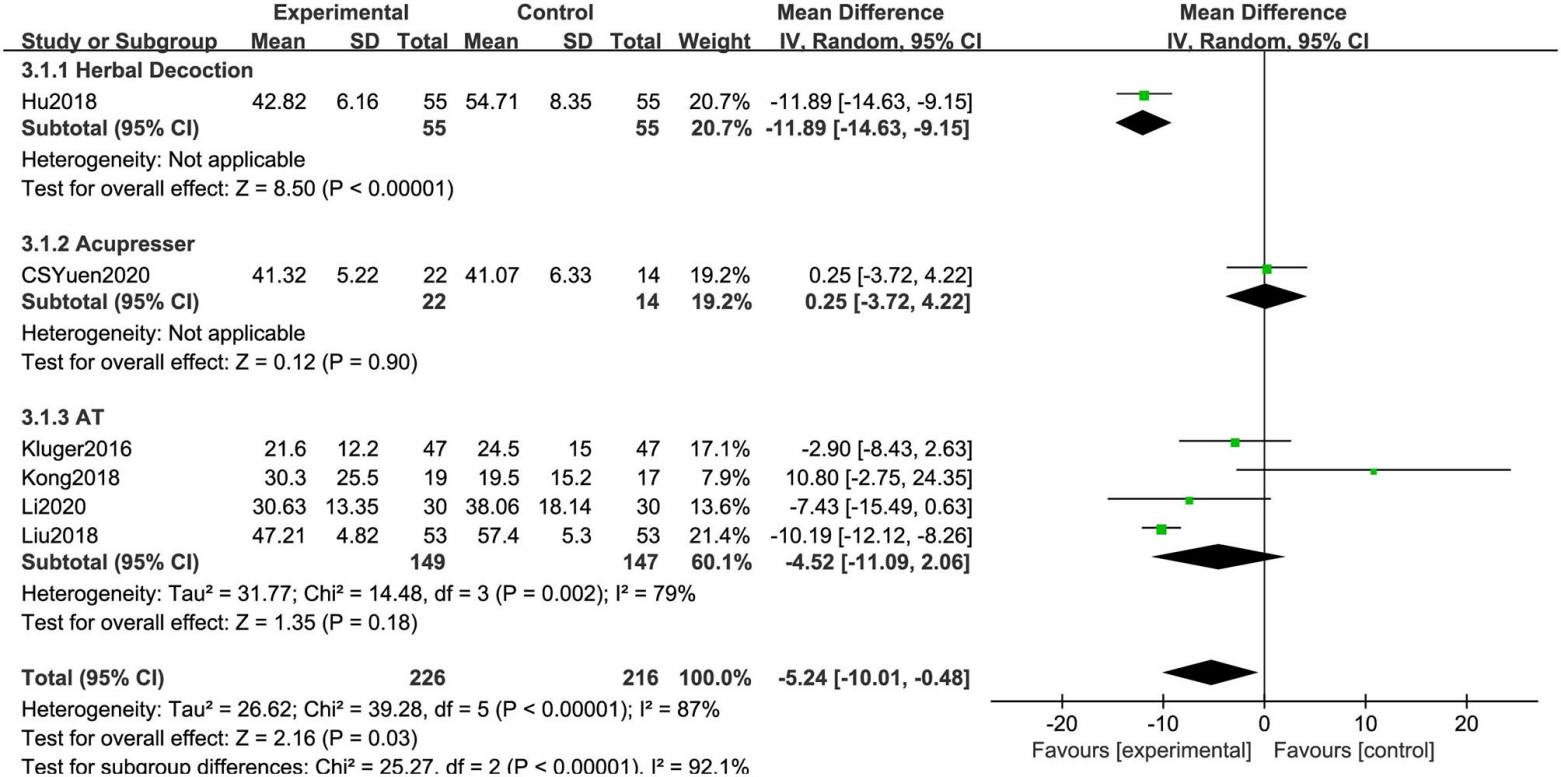
Comparison of acupuncture related therapies in terms of PDQ-39.

As shown in Figure 9, in six RCTs, the combination of acupuncture-related therapies and CM had a significantly greater effect than CM (MD: −5.24, 95% CI: −10.01 to −0.48, *P* = 0.03). The heterogeneity was significant (χ^2^ = 39.28, *P* < 0.01, *I*^2^ = 87%). Similarly, significant differences were observed among the subgroups (χ^2^ = 25.27, *P* < 0.01, *I*^2^ = 92.1%) (Figure 9).

##### Dosage of Madopar

**Figure 10:**
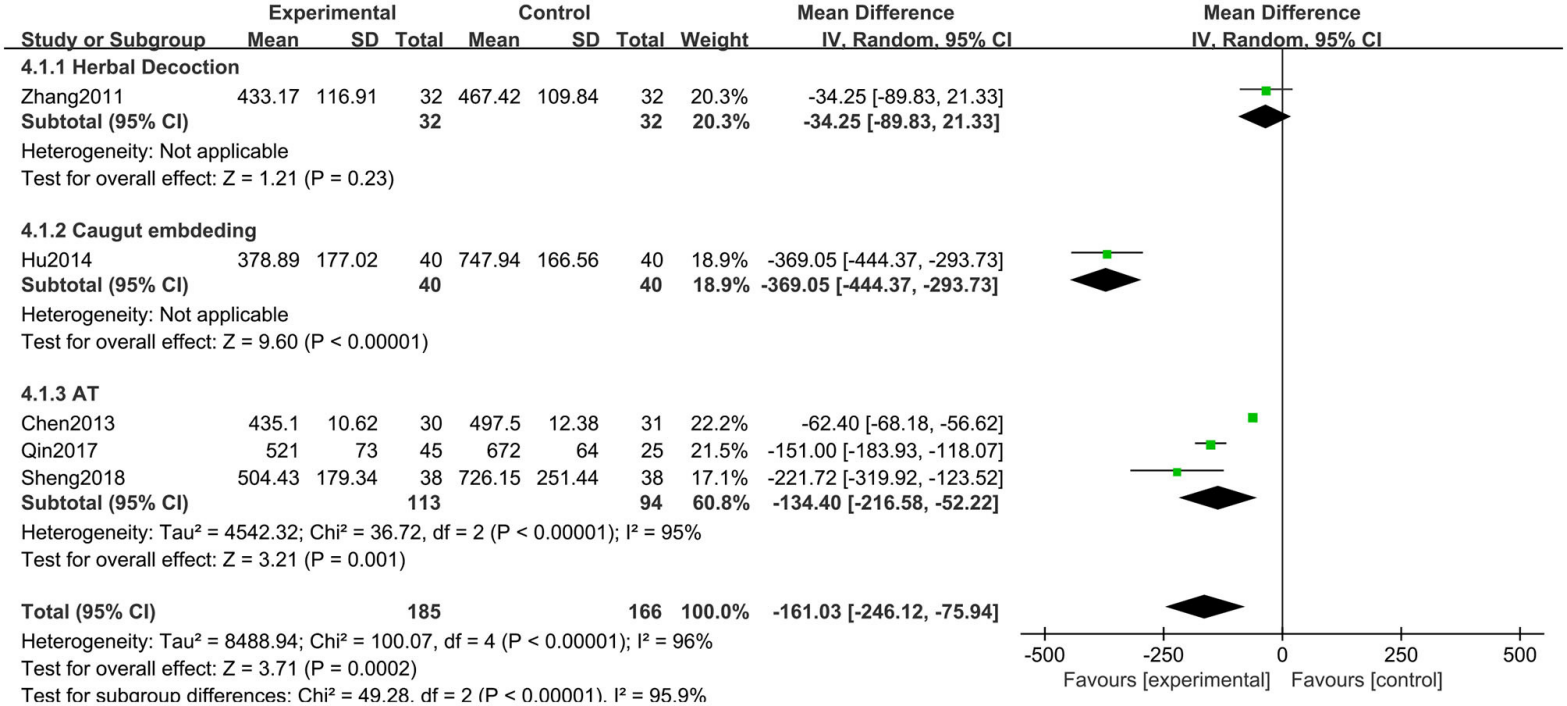
Comparison of acupuncture related therapies in terms of Dosage of Madopar (mg).

Five RCTs including 351 participants used the reductions in the dosage of Madopar to evaluate the treatment effects. The meta-analysis showed that the experimental group was significantly superior to the CM group (MD: −161.03, 95% CI: −246.12 to −75.94, *P* < 0.01). The heterogeneity was significant (χ^2^ = 100.07, *P* < 0.01, *I*^2^ = 96%). Similarly, significant differences were observed among the subgroups (χ^2^ = 49.28, *P* < 0.01, *I*^2^ = 95.9%) (Figure 10).

##### Hamilton Depression Scale

**Figure 11:**
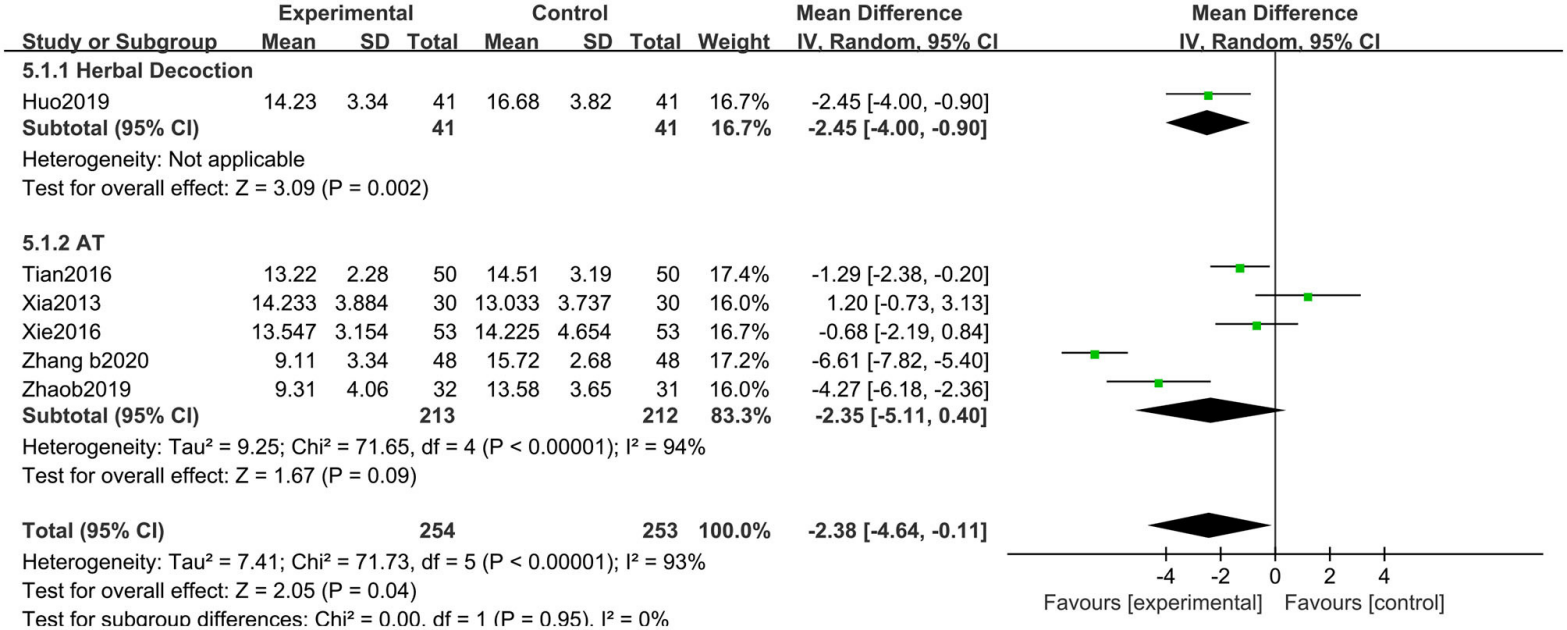
Comparison of acupuncture related therapies in terms of HAMD.

The HAMD (17 items) scores improved with acupuncture-related therapies combined with CM (MD: −2.38, 95% CIs: −4.64 to −0.11; *P* < 0.05). Heterogeneity existed across the trials (χ^2^ = 71.73, *P* < 0.01, *I*^2^ = 93%). No significant differences were observed among the subgroups (χ^2^ = 0.00, *P* = 0.95, *I*^2^ = 0%) (Figure 11).

##### Mini-Mental State Examination

**Figure 12:**
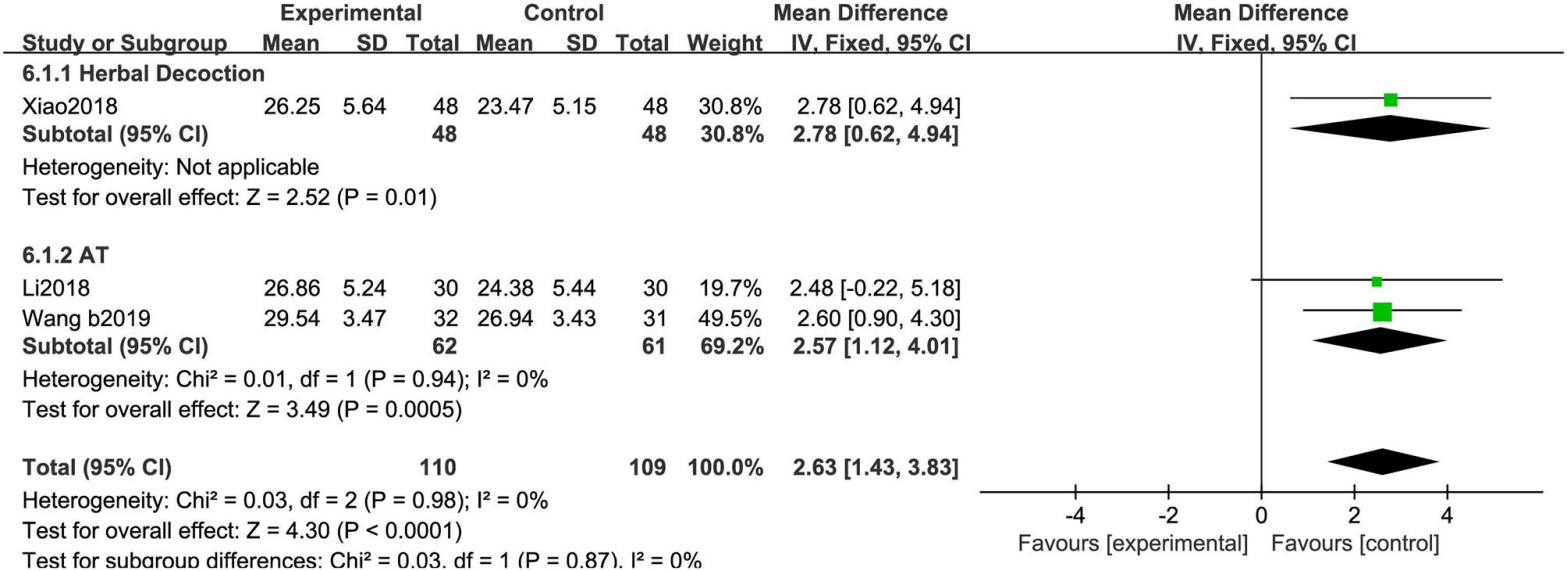
Comparison of acupuncture related therapies in terms of MMSE.

We saw a greater increase in MMSE scores in the experimental group than in the control group (MD: 2.63, 95% CIs: 1.43 to 3.83; *P* < 0.01). There was no heterogeneity across the trials (χ^2^ = 0.03, *P* = 0.98, *I*^2^ = 0%). No significant difference was observed between the two subgroups (χ^2^ = 0.03, *P* = 0.87, *I*^2^ = 0%) (Figure 12).

### Qualitative Analysis of Specific Methods

A qualitative analysis of specific methods of AT is described as follows.

#### Bee Venom Therapy

Since 2012, only three RCTs have studied the efficacy of bee venom therapy. The result of Cho's study (Cho et al., 2012) showed that the BVA group had significantly improved UPDRS-II, UPDRS-III, and UPDRS-total scores as well as improvements in several non-motor symptoms in both treatment groups after 8 weeks. After 6 years, Cho K. H. et al. (2018) performed another RCT and found that the AT+BVA group had significant improvements in the UPDRS-II+ III, UPDRS-II, UPDRS-III, and postural instability and gait disorder (PIGD) scores than the CM group (*P* < 0.01), but there was no evidence that the AT+BVA group was superior to the SA+saline group. In 2016, Hartmann et al. (2016) investigated the potential therapeutic and neuroprotective effects of BV injections, in comparison with saline placebo. No significant effects of BV were seen on UPDRS-III scores.

#### Acupoint Injection

One publication was found examining the short- and long-term benefits and safety of acupoint injections of kakkonein for PD (Liu et al., 2015). An RCT was conducted in which 135 participants were divided into three groups: (1) AT+Madopar; (2) acupoint injection+Madopar; and (3) Madopar only. The results showed that groups 1 and 2 were markedly superior to group 3 on UPDRS-I and UPDRS-II scores (*P* < 0.05). When groups 1 and 2 were compared, there was a non-significant difference on UPDRS-I scores, while the acupoint injection group showed better efficacy than the AT group on UPDRS-III scores (*P* < 0.05).

#### Abdominal Acupuncture

Only one RCT evaluated the efficacy of abdominal acupuncture for PD. Wen (Wen et al., 2008) observed the clinical effect of abdominal acupuncture plus moxibustion for the treatment of PD. They reported that the abdominal acupuncture plus moxibustion group showed better efficacy than the control group on UPDRS-II and UPDRS-total scores.

#### Self-Acupressure

Yuen et al. (2020) investigated the efficacy of conduction and self-acupressure (CE and SA) for improving PDQ-39 scores in PD patients. They found that CE and SA had the potential to treat non-motor symptoms, but inconclusive effectiveness based on PDQ-39 scores was found between the two groups.

#### Acupoint Catgut Embedding

Two articles evaluated the efficacy of catgut embedding alone or combined with other methods. Zhu (Zhu, 2016) evaluated the efficacy of catgut embedding for PD and found that catgut embedding plus CM had significant efficacy as compared with the CM group. Another publication (Hu et al., 2014) reported that catgut embedding in combination with herbal decoction plus Madopar compared with Madopar alone was significantly different based on UPDRS-I to III and UPDRS-total (*P* < 0.05).

#### Moxibustion

Five articles reported the efficacy of moxibustion [AT combined with moxibustion (60%) or moxibustion alone (40%)]. Evaluation of the total efficacy across all the articles (Zhang et al., 2005; Deng et al., 2014; Wu, 2014) yielded that the experimental group was superior to the control group. Wu (2014) mentioned that UPDRS-II scores could be improved by mild moxibustion (*P* < 0.05), but there was no evidence that the moxibustion group was superior to the control group on UPDRS-I, UPDRS-III, UPDRS-IV, and UPDRS-total scores. Shen et al. (2019) reported that UPDRS-II and UPDRS-III scores could be improved by scalp electroacupuncture combined with moxibustion plus CM. Shen et al. (2018) reported that the experimental group significantly improved based on BBS and PAC-QOL scores (*P* < 0.05), but there was no evidence that the moxibustion group was superior to the control group based on UPDRS-III scores.

#### Herbal Decoction

Six studies compared the effects of combined herbal medicine and AT, all of which found that herbal decoction combined with AT had a positive influence on PD symptoms (*P* < 0.05).

## Discussion

### Summary of Results

Several publications have indicated that acupuncture is widely and increasingly used as an adjuvant therapy in patients with PD. Previous reviews tended to discuss one type of acupuncture therapy, and most of them have discussed classical acupuncture (including scalp and electric acupuncture) on PD, but only a few referred to other types, such as BVA, catgut embedding, and moxibustion. This study differed in offering some important insights in discussing the various acupuncture-related therapies for PD and bringing all the evidence together from the numerous studies into one review to estimate the overall effect of the combination of acupuncture-related therapies with CM vs. CM alone. It also provided indirect comparisons of the different acupuncture methods used.

In the meta-analysis, it was suggested that the overall effect of acupuncture-related therapies combined with CM had significant improvements on nine outcomes: UPDRS-total, UPDRS-II, UPDRS-III, UPDRS-I, UPDRS-IV, PDQ-39, HAMD, Dosage of Madopar, and MMSE measures. For the primary outcomes, meaningful improvements were seen by MCIC. Subgroup difference tests of different acupuncture-related therapies used in the treatment of PD were implemented in this meta-analysis. Non-significant differences were identified on UPDRS-total, UPDRS-II, HAMD, and MMSE scores. In contrast, the outcomes regarding the UPDRS-III, UPDRS-I, UPDRS-IV, PDQ-39, and Dosage of Madopar showed differences in the treatment effects between the different interventions. However, since the data within each method were limited and these comparisons were based on indirect comparisons, the results should be interpreted with caution.

Qualitative analysis of specific methods indicated that they were all good candidates for future clinical evaluations, especially for non-motor symptoms of PD. Bee venom therapy and abdominal acupuncture are widely used in the clinic and have been suggested to be definitely effective (Awad et al., 2017). Regarding abdominal acupuncture, due to the painless procedure and effectiveness, it is easy to practice and worthy of clinical promotion (Chen et al., 2007). Meanwhile, self-acupressure can be promoted as a home-based therapy and conducive to relieving non-motor symptoms, especially in the gastrointestinal tract (Yuen et al., 2020). Moxibustion may be better for non-motor symptoms, especially for PD-related constipation. For depressive disorders associated with PD, classical AT proved useful for depressive disorders in several clinical trials (Yeung et al., 2011). Based on syndrome differentiation, clinicians may also prefer to use acupuncture combined with herbal decoction to treat PD-related depression. Moreover, compared with manual acupuncture, BVA, catgut embedding, and acupoint injection are all improved forms of classical acupuncture and are easy to promote due to the increased stimulation effect, longer lasting stimulation without additional biological effects, and lower expense (Zhu, 2016; Sheng et al., 2019).

### Adverse Events and Safety

Only 13 (19.70%) articles discussed adverse events, two (Cho et al., 2012; Xu et al., 2020) (3.03%) of which reported that there were no adverse events, and 12 (Jiang X. M. et al., 2006; Yang et al., 2006b; Chen et al., 2012; Cho et al., 2012; Hartmann et al., 2016; Kluger et al., 2016; Zhou et al., 2016; Kong et al., 2017; Cho S. Y. et al., 2018; Lin Z. C. et al., 2018; Xiao and Zhang, 2018; Yuen et al., 2020) (18.18%) publications reported the details of adverse events. The remaining two (Chen et al., 2013; Hu et al., 2014) (3.03%) studies reported that there was no significant change in biochemical parameters after treatment. No serious adverse events related to acupuncture-related therapies were reported.

### Mechanisms of Acupuncture-Related Therapies

All the included RCTs stated that the rationale for method selection was drawn from traditional CM (TCM) theory. Some hypotheses for the underlying mechanism of acupuncture in combination with Madopar have been proposed. Kluger summarized the following: increased glucose metabolism, basal ganglia-thalamocortical circuit activity, and reduced loss of dopaminergic neurons (Ghaffari and Kluger, 2014). Xiao suggested that acupuncture may work for PD in two ways: by changing the expression of some genes and proteins, we can recover the postsynaptic changes caused by dopamine loss and reactivate the brain circuit with dysfunction (Xiao, 2015).

BVA possesses neuroprotective potential in neurodegenerative diseases (Wehbe et al., 2019), and it is believed that antiapoptotic and anti-inflammatory effects are potential mechanisms (Ghaffari and Kluger, 2014). According to the results of Lu et al. 's study (Lu et al., 2019), moxibustion can effectively reduce the death of substantia nigra neurons, suppress ferroptosis, and increase tyrosine hydroxylase activity to protect neurons in rats with PD. Abdominal acupuncture is based on holographic medicine by acupuncture of the innate meridian system of the abdomen, which may directly stimulate the “gut-brain” and release neurotransmitters to act on target organs (Luo et al., 2008). Based on the theory of meridians and acupoints, catgut embedding acupuncture has longer-lasting stimulation on acupoints to improve the clinical effect. The mechanism of acupoint injection may be related to neurogenic inflammation of visceral tissue (Cao et al., 2001). Herbal medicine has been proven to be effective in PD based on syndrome differentiation (Wang et al., 2012). The combination of AT and herbal medicine can enhance the therapeutic effect, especially for PD-related complications.

### Implications

Our results are consistent with most previous reviews in that acupuncture-related therapies combined with CM as an adjunctive treatment can significantly improve motor and non-motor symptoms of PD. The early stage of PD is dominated by motor symptoms, while the later stage is plagued by non-motor symptoms more. Acupuncture-related therapy has been studied fairly advanced in improving fatigue, sleepiness, depression, constipation, and cognitive decline; and the effect is positive. However, subgroup difference tests of different acupuncture methods were also emphasized.

We discussed the clinically meaningful improvement by the MCIC. Schrag's study reported the threshold mean change in UPDRS scores. Their findings suggested a mean change of at least 2–3 points in UPDRS-II scores, and 5 points in UPDRS-III scores is meaningful (Schrag et al., 2006). Another study (Shulman et al., 2010) showed that changes of 2.5 to 5.2 points (2.5 points for minimal, 5.2 for moderate, and 10.8 for large) in UPDRS-III scores represented clinically meaningful differences. In our study, we found a −3.90 point improvement in UPDRS-III scores and −3.96 point improvement in UPDRS-II scores. We found that the total UPDRS scores in these two articles were a synthesis of UPDRS-I, UPDRS-II, and UPDRS-III rather than a total score of I to IV. Therefore, clinical improvements described in our article refer to UPDRS-II and UPDRS-III scores, and we cannot draw conclusions regarding UPDRS-total scores through MCIC.

Almost all patients with PD experience the deterioration of body function. With insidious onset and slow progression, PD patients need long-term management. Considering the complications associated with anti-Parkinson drugs, they will not choose to start early if PD symptoms did not interfere with their daily lives (Thanvi and Lo, 2004). Therefore, clinicians and researchers are eagerly seeking more strategies to increase the efficacy and reduce the side effects of anti-Parkinson drugs. Dosage of anti-Parkinson drug is a relevant and valid outcome measure. Long-term use of anti-Parkinson drugs will have many side effects, and with the progress of the disease, the amount of anti-Parkinson's drugs will gradually increase. Acupuncture-related therapies, based on TCM syndrome differentiation, can improve patients' symptoms from different angles and reduce medication, which is worth popularizing.

Merck (Marck et al., 2009) suggested that a multidisciplinary approach would appear to be preferable. This meta-analysis showed that there were non-significant differences in UPDRS-total, UPDRS-II, HAMD, and MMSE scores. For the secondary outcomes of the UPDRS-III, UPDRS-I, UPDRS-IV, PDQ-39, and Dosage of Madopar, we found differences between subgroups using different interventions, but we could not determine which method was more effective. Therefore, large and rigorous RCTs are recommended to directly compare the different methods, or network meta-analysis is needed further.

In general, this systematic review and meta-analysis could not provide prescriptions for individual treatment; instead, we hope to offer therapists a menu of treatment strategies and help them devise personalized interventions. Based on the results described above, therapists can choose the most suitable method for PD patients depending on the individual interests and complaints.

### Strengths and Limitations

The strengths of our article were as follows. Clinicians presently prefer to use different types of acupuncture to ameliorate the symptoms and complications of PD. We conducted a comprehensive search for different types of acupuncture-related therapies plus CM vs. CM. Studies with some methods, such as BVA, catgut embedding acupuncture, and acupoint injection, are still lacking, so we performed qualitative and quantitative analyses according to the available data. In the meantime, we performed a subgroup difference test of the different acupuncture therapies used for PD. Recently, an increasing number of clinicians and scientists have recognized that non-motor symptoms of PD are detrimental factors in the QOL of advanced PD patients (Asakawa et al., 2016). Previous reviews paid less attention to PD-related non-motor symptoms, such as fatigue, sleepiness, depression, constipation, and cognitive decline. In our article, we evaluate the total therapeutic efficacy and progression of PD by UPDRS-IV and UPDRS total scores. Since the UPDRS has the weakness of less emphasis on important non-motor symptoms (Movement Disorder Society Task Force on Rating Scales for Parkinson's Disease, 2003; Asakawa et al., 2016), we used the PDQ-39 to assess QOL, the HAMD to evaluate psychiatric symptoms, and the MMSE to further evaluate cognitive function.

There are some limitations in this review. First, the methodological quality of the included trials was generally unsatisfactory. A total of 59.09% of publications described the method of random allocation, and less than half of studies conducted allocation concealment and blinding of the participants and assessors. In the future, well-designed trials following the CONSORT guidelines are required. Second, it is possible that we may have missed relevant literature published in non-English or non-Chinese languages. Third, we only extracted data on immediate effects after treatment. We could not confirm the long-term effects because only a few articles provided data on long-term outcomes. PD is a chronic and progressive disease. Temporary benefits are not sufficient for disease management, and the long-term benefits of acupuncture-related therapies need to be explored (Cho K. H. et al., 2018). Fourth, with the exception for manual acupuncture, the number of included studies examining other acupuncture-related therapies was relatively small, resulting in limited power. Although cupping therapy and scrapping therapy belong to acupuncture-related therapies, we searched all the electronic databases, and we did not find sufficient evidence.

## Conclusion

To our knowledge, this is the first meta-analysis to investigate the effects of different types of acupuncture as an adjuvant for treating patients with PD. Acupuncture-related therapies in combination with CM were noted to be safe and feasible. In addition, our review emphasizes the differences between varieties of acupuncture therapies being used in PD. The meta-analysis demonstrated that there were non-significant differences between subgroups with different acupuncture methods on the UPDRS-total, UPDRS-II, HAMD, and MMSE scores. Taking into account the specialty of different acupuncture types and the complex symptoms of PD, we recommend that patients could have been treated with more intensive methods, resulting in great success in treating motor and non-motor symptoms.

Considering the limitations, especially the relatively small number of acupuncture-related therapies and the considerable heterogeneity among studies, our results should be interpreted with caution. Future large, randomized trials with strict methodological quality and long-term follow-up are warranted.

## Data Availability Statement

The original contributions presented in the study are included in the article/supplementary material, further inquiries can be directed to the corresponding author/s.

## Author Contributions

XPW wrote the manuscript. LML, KBL, and ZYW contributed to the conception. QW and SG search the literature. XPW, HW, and LML were involved in the data analysis. XPW, XLY, BJ, PGS, and CYW contributed to the acquisition of data. All authors have read and approved the final manuscript.

## Conflict of Interest

The authors declare that the research was conducted in the absence of any commercial or financial relationships that could be construed as a potential conflict of interest.
